# Oncogene-induced matrix reorganization controls CD8^+^ T cell function in the soft-tissue sarcoma microenvironment

**DOI:** 10.1172/JCI167826

**Published:** 2024-04-23

**Authors:** Ashley M. Fuller, Hawley C. Pruitt, Ying Liu, Valerie M. Irizarry-Negron, Hehai Pan, Hoogeun Song, Ann DeVine, Rohan S. Katti, Samir Devalaraja, Gabrielle E. Ciotti, Michael V. Gonzalez, Erik F. Williams, Ileana Murazzi, Dimitris Ntekoumes, Nicolas Skuli, Hakon Hakonarson, Daniel J. Zabransky, Jose G. Trevino, Ashani Weeraratna, Kristy Weber, Malay Haldar, Joseph A. Fraietta, Sharon Gerecht, T.S. Karin Eisinger-Mathason

**Affiliations:** 1 Abramson Family Cancer Research Institute, Department of Pathology and Laboratory Medicine, Penn Sarcoma Program, University of Pennsylvania Perelman School of Medicine, Philadelphia, Pennsylvania, USA.; 2 Department of Chemical and Biomolecular Engineering, Institute for NanoBioTechnology, Johns Hopkins University, Baltimore, Maryland, USA.; 3 Children’s Hospital of Philadelphia, Philadelphia, Pennsylvania, USA.; 4 Department of Microbiology, Center for Cellular Immunotherapies, Parker Institute for Cancer Immunotherapy, University of Pennsylvania Perelman School of Medicine, Philadelphia, Pennsylvania, USA.; 5 Department of Biomedical Engineering, Duke University, Durham, North Carolina, USA.; 6 Department of Oncology, The Sidney Kimmel Cancer Center, Johns Hopkins School of Medicine, Baltimore, Maryland, USA.; 7 Division of Surgical Oncology, Department of Surgery, Virginia Commonwealth University School of Medicine, Richmond, Virginia, USA.; 8 Department of Biochemistry and Molecular Biology, Johns Hopkins Bloomberg School of Public Health, Baltimore, Maryland, USA.; 9 Department of Orthopaedic Surgery, Penn Sarcoma Program, University of Pennsylvania Perelman School of Medicine, Philadelphia, Pennsylvania, USA.

**Keywords:** Oncology, Cancer immunotherapy, Extracellular matrix, Skeletal muscle

## Abstract

CD8^+^ T cell dysfunction impedes antitumor immunity in solid cancers, but the underlying mechanisms are diverse and poorly understood. Extracellular matrix (ECM) composition has been linked to impaired T cell migration and enhanced tumor progression; however, impacts of individual ECM molecules on T cell function in the tumor microenvironment (TME) are only beginning to be elucidated. Upstream regulators of aberrant ECM deposition and organization in solid tumors are equally ill-defined. Therefore, we investigated how ECM composition modulates CD8^+^ T cell function in undifferentiated pleomorphic sarcoma (UPS), an immunologically active desmoplastic tumor. Using an autochthonous murine model of UPS and data from multiple human patient cohorts, we discovered a multifaceted mechanism wherein the transcriptional coactivator YAP1 promotes collagen VI (COLVI) deposition in the UPS TME. In turn, COLVI induces CD8^+^ T cell dysfunction and immune evasion by remodeling fibrillar collagen and inhibiting T cell autophagic flux. Unexpectedly, collagen I (COLI) opposed COLVI in this setting, promoting CD8^+^ T cell function and acting as a tumor suppressor. Thus, CD8^+^ T cell responses in sarcoma depend on oncogene-mediated ECM composition and remodeling.

## Abstract 2

In the sarcoma microenvironment, oncogenic YAP1 promotes collagen VI deposition, which opposes and reorganizes collagen I, compromises CD8+ T cell function, and impairs anti-tumor immunity.

## Introduction

Immunosuppression in the solid tumor microenvironment (TME) impedes T cell–mediated antitumor immunity. Tumors evade host adaptive immune responses by inducing CD8^+^ T cell dysfunction, a hypofunctional state characterized by overexpression of inhibitory cell surface receptors (e.g., PD-1, TIM-3, LAG3), reduced effector function, and impaired proliferative capacity (1). Mechanisms underlying CD8^+^ T cell dysfunction in solid cancers are of significant interest due to their impact on immunotherapy strategies. However, most studies in this field have focused on the roles of continuous antigen exposure/repetitive T cell receptor (TCR) stimulation, immune checkpoint–mediated inhibitory signaling, and immunosuppressive cytokines (2). Moreover, the importance of TME contexture in the setting of T cell–based therapies is poorly described. Thus, a more comprehensive and physiological evaluation of CD8^+^ T cell dysfunction in solid tumors is critical for improving our understanding of immune evasion mechanisms in the TME and advancing actionable interventions.

Soft-tissue sarcomas (STSs) are heterogeneous solid mesenchymal tumors with approximately 70 distinct histologic subtypes (3). These lesions are characterized by mesenchymal gene expression, extensive extracellular matrix (ECM) deposition, and increased stiffness relative to normal tissues (4–6). Interestingly, these features are also observed in high-grade, poorly differentiated epithelial tumors, where they are linked to progression, therapeutic resistance, and poor clinical outcomes (7). Recent studies have shown that the ECM facilitates cancer progression in part by inhibiting T cell migration/infiltration (8–11). However, the roles of individual ECM proteins in this process are only beginning to be defined. Moreover, little research has addressed the effects of ECM molecules on T cell function in solid tumors or identified upstream regulators of aberrant ECM deposition in this context. This paucity of available data indicates that further study, particularly in vivo, is necessary.

The 28 distinct molecular species of the collagen superfamily are some of the most abundant and diverse ECM constituents in normal and malignant tissues (12). Although the roles of specific collagen species in cancer-associated processes are ill-defined, a growing body of literature indicates that they can have context-specific functions in the TME. For example, type I collagen (ColI), a “prototypical” fibrillar collagen, promotes or is associated with malignant progression in some settings but has antitumor effects in others (13–17). These findings underscore the need to systematically interrogate the roles of individual collagens in specific tumor contexts, particularly with respect to their potential impacts on adaptive immunity.

Undifferentiated pleomorphic sarcoma (UPS) is a relatively common STS subtype that predominantly arises in adult skeletal muscle and has a 10-year survival rate of only about 25% (3, 18). Although some STSs are considered immunologically “cold,” UPS patients have exhibited objective clinical responses to immune checkpoint inhibition in recent clinical trials (19, 20). These encouraging findings suggest that studies of UPS may provide valuable insights into strategies for enhancing T cell function and immunotherapy responses in solid tumors. Our previous work linked the intrinsic oncogenic functions of the transcriptional coregulator Yes-associated protein 1 (YAP1), the central Hippo pathway effector, to UPS cell proliferation, tumor growth, and reduced human patient survival (21–24). However, we had not investigated the contribution of YAP1 to the UPS TME or immune cell activity. In some carcinomas, cancer cell–intrinsic YAP1 modulates macrophage and myeloid-derived suppressor cell recruitment and differentiation, suggesting an immunomodulatory role (25, 26). However, this observation has not been confirmed in mesenchymal cancers. YAP1 also possesses mechanosensory functions, and its nuclear localization and activity increase in response to stiff environments such as those in tumor tissue (27). Therefore, herein, we interrogated the role of UPS cell–intrinsic YAP1 in the regulation of ECM deposition/organization and adaptive immune cell function in the TME. We discovered that YAP1 regulates ECM composition and cytotoxic T cell function, and that collagen type VI (ColVI), a microfibrillar collagen, indirectly modulates effector T cell function by opposing and remodeling ColI. We further identify COLVI as a putative diagnostic and survival biomarker in human UPS. Our findings implicate YAP1 inhibition, in combination with immunotherapy, as a promising approach to mitigate immune evasion in the sarcoma TME.

## Results

### UPS cell–intrinsic Yap1 inhibits T cell activation and promotes CD8^+^ T cell dysfunction.

**Figure 1:**
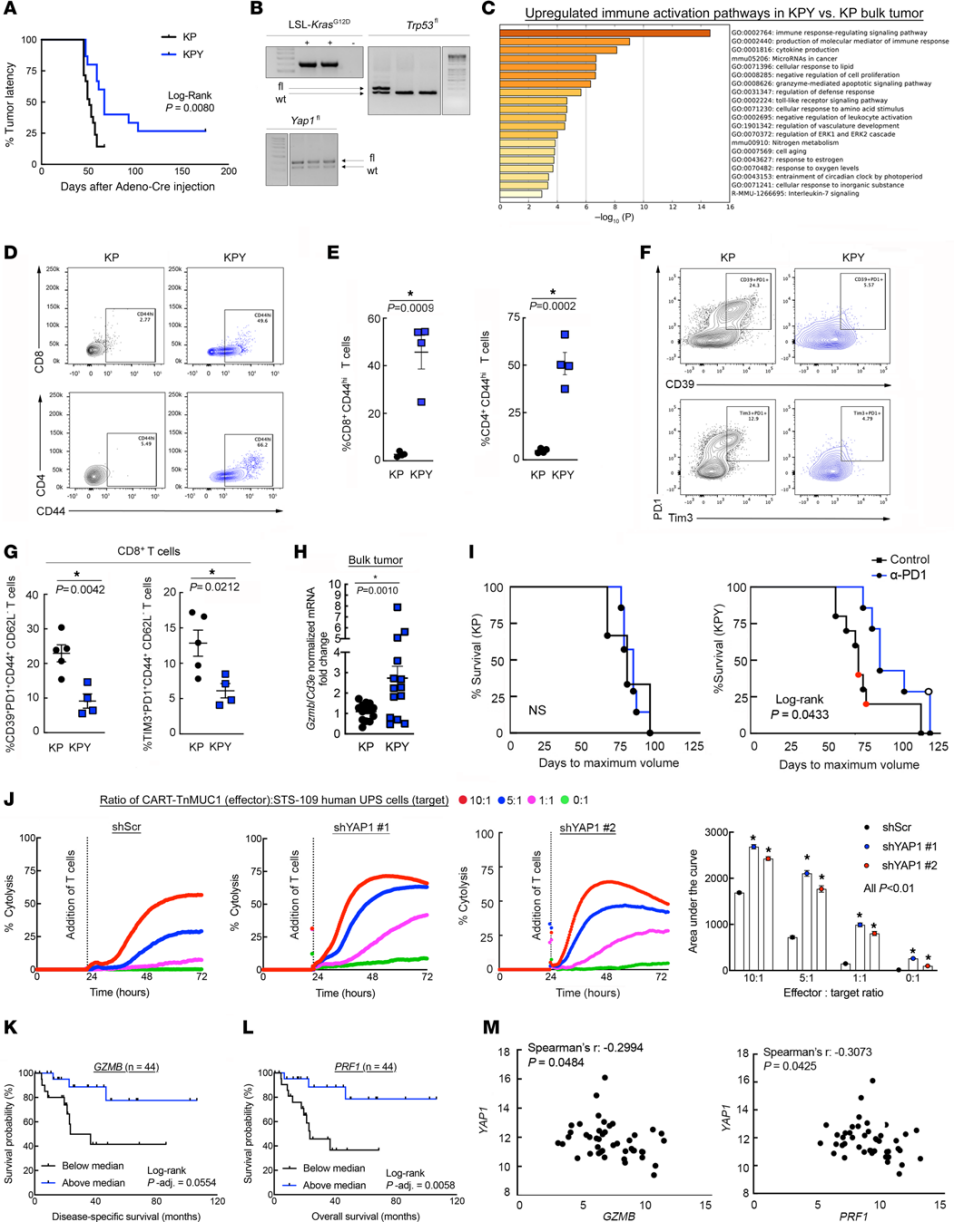
YAP1^+^ UPS cells inhibit CD8^+^ T cell activation and promote dysfunction. (**A**) Kaplan-Meier latency curves of KP and KPY UPS tumors (*n* > 10 per genotype). (**B**) Validation of genotypes from **A** (fl, floxed; wt, wild type). The *Trp53^fl^* and *Yap1^fl^* bands and their respective ladders are from the same gels but were separated for presentation. (**C**) Metascape pathway analysis of 5 unique bulk KP and KPY tumors. Includes all genes with greater than 2-fold expression increase in KPY versus KP, identified via microarrays. (**D** and **E**) Representative contour plots (**D**) and quantification (**E**) of CD8^+^CD44^hi^ and CD4^+^CD44^hi^ T cells in KP and KPY tumors. (**F** and **G**) Representative contour plots (**F**) and quantification (**G**) of CD39, Tim-3, and Pd1 expression in CD8^+^ T cells from KP and KPY tumors. For **E** and **G**, points represent individual tumors; 2-tailed unpaired *t* tests. (**H**) *Gzmb* quantitative reverse transcriptase PCR (qRT-PCR) in bulk KP and KPY tumors; 2-tailed unpaired *t* test. (**I**) Kaplan-Meier survival curves of KP and KPY mice treated with α-Pd1 or control. Red and black circles in control curves indicate IgG-injected and uninjected mice, respectively. Open circle, mouse with durable tumor regression; *x* axis, days to maximum volume since adeno Cre injection. (**J**) Average longitudinal cytolysis of shScr or shYAP1 human STS-109 UPS cells during coculture with CART-TnMUC1 cells from 3 independent human donors. Measurements indicate percent target (UPS) cell cytolysis. Quantification: 1-way ANOVA with Dunnett’s test (vs. shScr) for each ratio. Points for individual replicates overlap. shScr data are identical to those in Figure 3, D and E (performed in the same experiment). (**K** and **L**) Kaplan-Meier survival curves of UPS patients in TCGA-SARC stratified by intratumoral *GZMB* (**K**) and *PRF1* (**L**) expression. (**M**) Correlation of *YAP1* with *GZMB* and *PRF1* gene expression in UPS tumors from TCGA-SARC.

Using the genetically engineered mouse model (GEMM) of skeletal muscle UPS, *Kras^G12D/+^* *Trp53^fl/fl^* (KP) (28, 29), we previously showed that UPS cell–intrinsic Yap1 promotes tumorigenesis and progression by activating NF-κB (22). In this system, tumors are generated by injection of adenovirus-expressing Cre recombinase into the gastrocnemius muscle. Recombination initiates oncogenic *Kras* expression and deletes *Yap1^fl^* alleles in infected muscle progenitor cells (28, 29). *TP53* mutations and deletion are prevalent in human UPS (30), as is MAPK hyperactivation downstream of KRAS (31). Consistent with our previous work (22), we observed significantly increased tumor latency and similar rates of tumor development when we introduced *Yap1^fl/fl^* alleles into the KP GEMM, creating *LSL*-*Kras^G12D/+^* *Trp53^fl/fl^* *Yap1^fl/fl^* (KPY) animals (Figure 1, A and B, and Supplemental Figure 1A; supplemental material available online with this article; https://doi.org/10.1172/JCI167826DS1).

Our previous work has focused on mechanisms by which Yap1 impacts sarcoma cell–autonomous signaling and phenotypes such as proliferation, differentiation, and metastasis (21–23). Therefore, herein, we tested the hypothesis that UPS cell–intrinsic Yap1 also impacts the TME. To identify potential mechanisms of Yap1-mediated TME modulation, we explored a publicly available gene expression microarray data set previously published by our group (22) comparing 5 unique KP and KPY bulk tumors. Loss of *Yap1* enhanced expression of numerous immune activation pathways, suggesting that UPS cell–intrinsic Yap1 is immunosuppressive (Figure 1C). To investigate how Yap1 controls immunosuppression in UPS, we performed flow cytometric and immunohistochemical (IHC) analyses of KP and KPY tumors. We did not detect changes in myeloid cell infiltration or polarization, or differences in B cell content (Supplemental Figure 1, B–D). However, we did observe increased proportions of CD44^hi^CD8^+^ and CD44^hi^CD4^+^ T cells in KPY relative to KP tumors, indicating enhanced T cell activation (Figure 1, D and E). Furthermore, the percentage of dysfunctional effector CD8^+^ T cells (CD39^+^Pd1^+^ and Tim-3^+^Pd1^+^) was higher in KP tumors versus KPY (Figure 1, F and G). Markers of central CD8^+^ memory T cell differentiation (CD62L, CD127) remained unchanged (Supplemental Figure 2A). Importantly, in KPY mice, the observed increases in T cell activation could not be attributed to reduced immunosuppressive Foxp3^+^ regulatory T cell content, nor to enhanced T cell infiltration (Supplemental Figure 1, B–D). In fact, CD4^+^ and CD8^+^ T cell content was modestly decreased in KPY relative to KP tumors. Therefore, Yap1 may promote CD8^+^ T cell dysfunction but likely does not impact T cell recruitment to the TME.

To further explore the relationship between Yap1^+^ UPS cells and T cell activation, we evaluated levels of granzyme B (*Gzmb*), a T cell cytolysis marker, in GEMM tumors (Figure 1H). *Gzmb* expression (normalized to total T cells; *Cd3e*) was significantly increased in KPY versus KP tumors, further demonstrating that Yap1^+^ UPS cells are associated with immunosuppression. Therefore, to determine whether UPS cell–intrinsic Yap1 modulates T cell effector function in addition to inhibitory receptor expression, we treated tumor-bearing KP and KPY mice with anti-Pd1 or isotype control antibody. We hypothesized that immune checkpoint blockade would show increased efficacy in KPY animals due to enhanced T cell activation but have no effect in KP mice. Consistent with this hypothesis, time to maximum tumor volume was significantly increased in KPY animals, but not KP (Figure 1I). Notably, one KPY mouse experienced complete, durable tumor regression. We further evaluated the effect of YAP1^+^ UPS cells on T cell function with human chimeric antigen receptor T (CART) cells that target the Tn glycoform of mucin 1 (TnMUC1-CART cells) (32). This neoantigen is expressed on human STS-109 cells, derived from a UPS patient tumor (Supplemental Figure 2B). We cocultured TnMUC1-CART cells with STS-109 cells expressing control or *YAP1-*specific shRNAs (shYAP1) at multiple effector:target ratios and analyzed longitudinal cytolysis (Figure 1J and Supplemental Figure 2C). T cell cytolysis was enhanced in the presence of *YAP1-*deficient UPS cells, confirming that YAP1*^+^* UPS cells promote immunosuppression. To explore these data in a clinical context, we leveraged The Cancer Genome Atlas Sarcoma (TCGA-SARC) data set (30). Consistent with our experimental findings, gene expression levels of T cell cytolysis markers (*GZMB* and perforin [*PRF1*]) in human UPS tumors were associated with improved survival (Figure 1, K and L, and Supplemental Figure 2D) and negatively correlated with *YAP1* levels (Figure 1M). Thus, although some sarcomas are considered immunologically “cold,” our data suggest that cytotoxic T cell activation is a critical factor in UPS patient survival, and that modulating Yap1 and T cell activity may improve clinical outcomes.

### UPS cell–intrinsic Yap1 promotes collagen VI deposition in the TME.

We next sought to define the mechanism of crosstalk between Yap1^+^ UPS cells and infiltrating CD8^+^ T cells. Given recent studies in epithelial tumors showing that Yap1 can influence the cancer cell “secretome” (26, 33), we measured 31 cytokines and chemokines in sample supernatants from our CART cell cytolysis assays (Figure 1J). Many analytes were below the assay’s lower limit of detection, but those we could detect were generally stable across samples (Supplemental Figure 3, A and B). These findings suggest that YAP1 likely does not control CD8^+^ T cell function in the TME via cytokines or chemokines; thus, we focused on other potential mechanisms.

**Figure 2:**
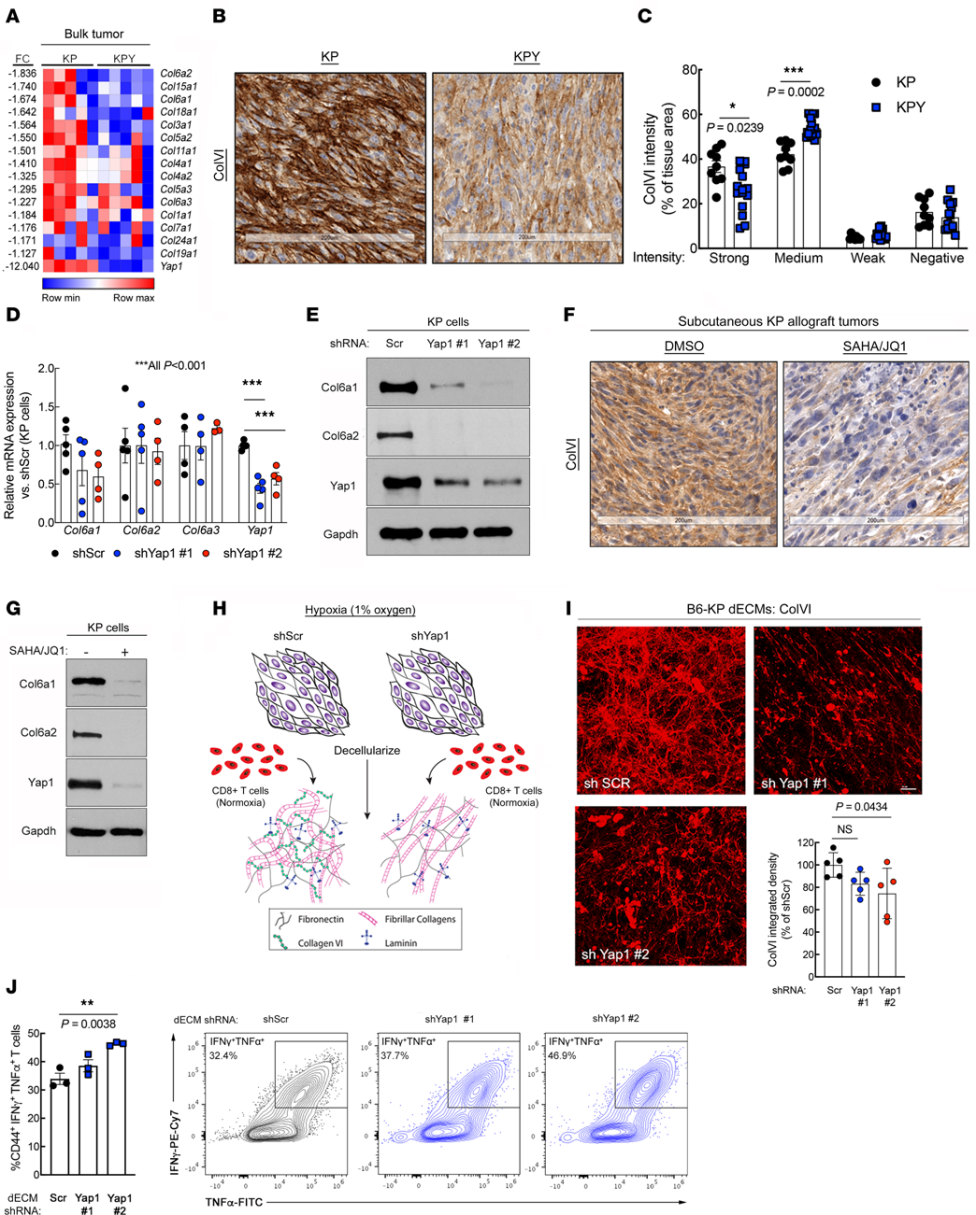
UPS cell–intrinsic Yap1 mediates ColVI deposition in the TME. (**A**) Heatmap of gene expression microarray data comparing 5 unique KP and KPY bulk tumors. The top one-third of collagen-encoding genes modulated by *Yap1* deletion are displayed. Supplemental Figure 3D shows the remaining two-thirds of collagen-encoding genes. FC, fold change. (**B** and **C**) Representative images (**B**) and quantification (**C**) of ColVI IHC in KP and KPY tumors; 2-tailed unpaired *t* tests with Welch’s correction and Holm-Šidák multiple-comparison test (*n* = 3–5 mice per genotype with 3 sections per mouse). Scale bars: 200 μm. (**D**) qRT-PCR of *Col6a1*, *Col6a2*, *Col6a3*, and *Yap1* gene expression in KP cells expressing a control or one of multiple independent *Yap1*-targeting shRNAs; 1-way ANOVA with Dunnett’s test (vs. shScr) for each gene. (**E**) Representative immunoblot of KP cells treated as in **D**. (**F**) Representative images of ColVI IHC in KP tumor–bearing mice treated with 25 mg/kg SAHA plus 50 mg/kg JQ1 or vehicle control for 20 days. Quantification is in Supplemental Figure 3M. Scale bars: 200 μm. (**G**) Representative immunoblot of KP cells treated with SAHA (2 μM) plus JQ1 (0.5 μM) or vehicle control for 48 hours. (**H**) Schematic of experimental model to assess immunomodulatory role of UPS cell–derived decellularized extracellular matrix (dECM). (**I**) Representative wide-field images and quantification of ColVI deposition in dECM from KP cells expressing control or *Yap1*-targeting shRNAs; 1-way ANOVA with Dunnett’s test (vs. shScr). Scale bar: 25 μm. Image brightness and contrast were adjusted for publication. (**J**) Quantification and representative contour plots showing IFN-γ and TNF-α coexpression in CD44^+^CD8^+^ T cells incubated on dECMs from control and shYap1-expressing KP cells. Each point represents T cells isolated from an individual mouse. One-way ANOVA with Dunnett’s test (vs. shScr). The shScr plot and data are identical to those in Figure 5A (performed in the same experiment).

YAP1 is a known modulator of mechanosensing properties associated with ECM remodeling (34). Therefore, we investigated whether ECM-related processes underlie YAP1-mediated T cell suppression in the UPS TME. Using our microarray data set of KP and KPY tumors to identify Yap1-dependent matrix genes, we found that many ECM- and tissue remodeling–associated pathways were altered in KPY tumors relative to KP (Supplemental Figure 3C). We also observed that genes encoding many members of the collagen superfamily, particularly collagen type VI (ColVI; e.g., *Col6a1*, *Col6a2*, *Col6a3*), were downregulated in KPY relative to KP tumors (Figure 2A and Supplemental Figure 3D). Quantitative reverse transcriptase PCR and IHC analysis of bulk tumor specimens revealed that KPY tumors exhibited a trend toward reduced ColVI deposition overall (Figure 2, B and C, and Supplemental Figure 3, E–H). We did observe some heterogeneity in expression, potentially due to ColVI secretion by multiple cell types including macrophages (35) and UPS cells themselves; however, IHC analysis clearly showed that KPY tumors exhibited significantly less strong-positive (3+) and significantly more moderately positive (2+) staining than KP tumors (Figure 2, B and C). We validated these findings in vitro with UPS cell lines derived from multiple unique KP GEMM tumors (KP230 and SKPY42.1 cells, referred to hereafter as “KP cells”). Specifically, KP cells transduced with one of multiple *Yap1*-specific shRNAs expressed substantially less Col6a1 and Col6a2 than control cells (Figure 2, D and E). In contrast, we could not validate a role for Yap1 in the modulation of collagen type III expression (*Col3a1*; Supplemental Figure 3I), nor that of other matrix genes such as fibronectin (*Fn1*; Supplemental Figure 3J), indicating the potential specificity of this regulation.

To confirm the relationship between Yap1 and ColVI in UPS with a pharmacologic approach, we treated tumor-bearing KP mice and KP cells in vitro with the histone deacetylase inhibitor (HDACi) vorinostat, also known as suberoylanilide hydroxamic acid (SAHA), and the BRD4 inhibitor JQ1, or vehicle control. We and others have reported that JQ1/SAHA (or other HDACi) combination treatment inhibits Yap1 expression in UPS and other cancers (22, 36, 37). These studies confirmed that ColVI gene and protein expression were substantially downregulated in SAHA/JQ1–treated cells and tumors (Figure 2, F and G, and Supplemental Figure 3, K–M). Together, these data support the conclusion that UPS cell–intrinsic Yap1 promotes aberrant ColVI deposition in the TME, whereas genetic and nonspecific pharmacologic inhibition of Yap1 can reverse this process.

As a transcriptional coactivator, Yap1 lacks a DNA-binding domain and must interact with Tea domain (TEAD) transcription factors to stimulate gene expression; Tead1 is enriched in skeletal muscle tissue (38) and potentially muscle-derived tumors. Therefore, to explore the mechanism by which Yap1 promotes ColVI deposition, we leveraged publicly available Tead1 ChIP-Seq data (Gene Expression Omnibus [GEO] GSE55186) from Yap1-driven embryonal rhabdomyosarcoma (eRMS) (39), skeletal muscle–derived tumors that lie on morphologic and transcriptional continua with UPS (40). In murine Yap1-driven eRMS (39), Tead1-ChIP signal was enriched in a region that overlapped with the *Col6a1* 5′-untranslated region (UTR), likely corresponding to the *Col6a1* promoter (Supplemental Figure 4A). A second peak approximately 5 kb upstream of the *Col6a1* 5′-UTR was also observed, potentially representing an enhancer region. Similarly, in cultured human eRMS cells (RD cells), TEAD1-ChIP signal was enriched approximately 9 kb upstream of the *COL6A1* 5′-UTR (Supplemental Figure 4B). These data suggest that transcriptionally active Yap1 upregulates ColVI deposition in skeletal muscle–derived sarcomas by directly stimulating *Col6a1* transcription.

### Yap1-mediated ColVI deposition promotes CD8^+^ T cell dysfunction.

Based on our findings, we hypothesized that Yap1-mediated ColVI deposition in the UPS TME promotes CD8^+^ T cell dysfunction. To test this idea, we developed a system wherein C57BL/6 KP cells (B6-KP cells) were seeded at 90% confluence and cultured under hypoxic conditions (1% O_2_), stimulating them to deposit ECM. This ECM was then decellularized (decellularized ECM; dECM) and incubated with activated syngeneic C57BL/6 CD8^+^ T cells (splenocytes) under normoxic conditions (21% O_2_) (Figure 2H). dECMs were generated under hypoxic conditions because it is well established that hypoxia stimulates robust ECM gene/protein expression and matrix remodeling in the TME (4, 7). Indeed, ColVI deposition was significantly increased in dECMs generated under hypoxia versus normoxia (Supplemental Figure 4, C and D). Thus, UPS dECMs generated under hypoxic conditions were used in all experiments given our focus on Yap1-mediated ECM deposition and not the role of hypoxia versus normoxia per se. Subsequent CD8^+^ T cell culture on dECMs was conducted under normoxic conditions given previous reports that hypoxia can either enhance or suppress CD8^+^ T cell expansion and function depending on tissue/experimental context and extent of TCR stimulation (41–44).

To determine whether Yap1-mediated ColVI deposition in UPS enhances T cell dysfunction, we generated dECMs from control and *Yap1*-deficient B6-KP cells. ColVI deposition was somewhat heterogeneous, but generally decreased in *Yap1-*deficient dECMs compared with controls, confirming the regulatory role of UPS cell–intrinsic Yap1 in ColVI secretion (Figure 2I). Although the observed reductions in ColVI were modest, these results were unsurprising, because culturing of cells at high confluence — which is required for matrix deposition in our system — is a well-established Yap1 suppressor (45) and can thereby minimize differences in Yap1 activity between shScr and shYap1 UPS cells. We cultured syngeneic CD8^+^ T cells on these dECMs and measured the expression of T cell inhibitory receptors by flow cytometry. The proportion of CD8^+^ T cells coexpressing Pd1 and Tim-3 was modestly reduced following culture on dECMs from *Yap1*-deficient compared with control UPS cells (Supplemental Figure 4, E and F). We also observed significantly higher percentages of CD8^+^ T cells coexpressing the cytolytic markers IFN-γ and TNF-α following culture on dECMs from shYap1 cells (Figure 2J), consistent with the results of our TnMUC1-CART assay (Figure 1J). Together, these results support the conclusion that UPS cell–intrinsic Yap1 promotes CD8^+^ T cell dysfunction.

**Figure 3:**
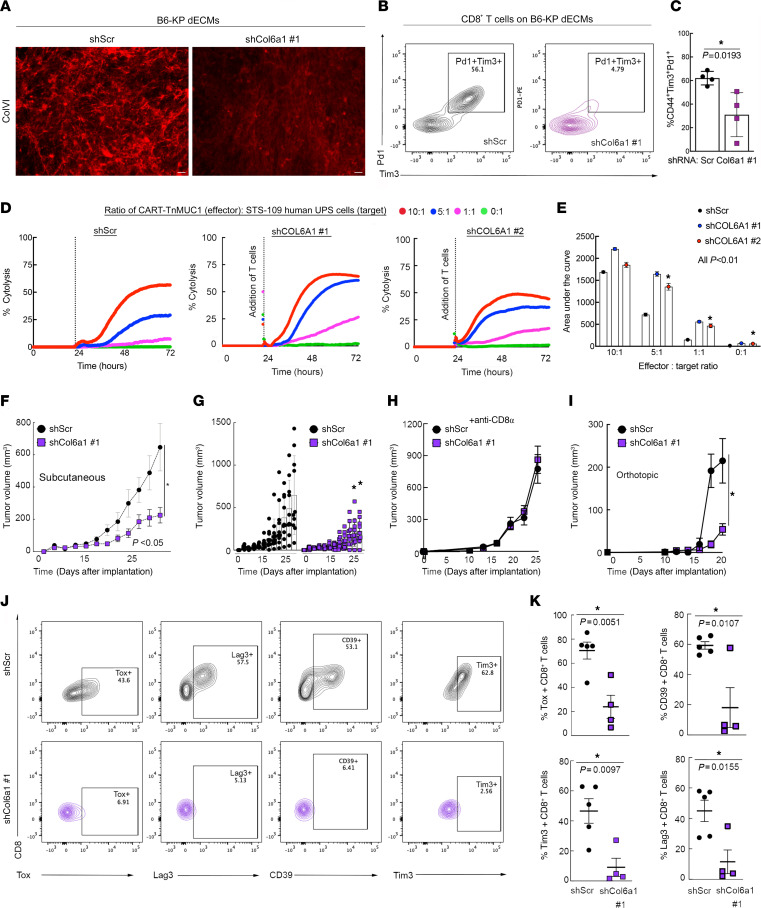
ColVI in the UPS TME promotes CD8^+^ T cell dysfunction. (**A**) Representative wide-field images of ColVI immunofluorescence in dECMs generated from control and shCol6a1-expressing B6-KP cells. Scale bars: 20 μm. Brightness and contrast were adjusted for publication. (**B** and **C**) Representative contour plots (**B**) and quantification (**C**) of Pd1 and Tim-3 coexpression in CD44^+^CD8^+^ T cells incubated on dECM derived from control or shCol6a1 KP cells. (**D**) Average longitudinal cytolysis of shScr- or shCOL6A1-expressing human STS-109 UPS cells cocultured with CART-TnMUC1 cells from 3 independent human donors (*n* = 2 for shCOL6A1 #1). Points for individual replicates overlap. Measurements indicate percent target cell (UPS cell) cytolysis. (**E**) Quantification of area under the curve from **D**; 1-way ANOVA with Dunnett’s test (vs. shScr) for each ratio. In **D** and **E**, shScr data are identical to those in Figure 1J (performed in the same experiment). (**F**) Tumor growth curves from subcutaneous (flank) syngeneic transplant of 3 × 10^4^ B6-KP cells in Matrigel expressing control or *Col6a1*-targeting shRNAs in syngeneic C57BL/6 mice; 2-way ANOVA. (**G**) Individual tumors from **F**. (**H**) Tumor growth curves depicting subcutaneous (flank) syngeneic transplant of 5 × 10^5^ KP cells (SKPY42.1 cell line) expressing control or *Col6a1*-targeting shRNAs in C57BL/6 mice treated with α-CD8α every 3 days. (**I**) Tumor growth curves depicting syngeneic orthotopic transplant (into the gastrocnemius muscle) of 2.5 × 10^5^ KP cells (SKPY42.1 cell line) expressing control or *Col6a1*-targeting shRNAs in C57BL/6 mice; 2-way repeated-measures ANOVA, SEM. (**J** and **K**) Representative contour plots (**J**) and quantification (**K**) of T cell dysfunction markers in CD8^+^ T cells from control and shCol6a1 orthotopic tumors from **I**. Each point in **K** represents an individual tumor. Two-tailed unpaired *t* tests.

We then determined the specific effects of ColVI, downstream of Yap1, on CD8^+^ T cell surface marker expression by generating dECMs from ColVI-deficient KP cells (Figure 3A). In this assay, we targeted *Col6a1*, rather than other ColVI-encoding genes, because *Col6a1* is indispensable for ColVI protein synthesis (46). *Col6a1* depletion in KP cells significantly reduced CD8^+^ T cell dysfunction in this assay, measured by Pd1/Tim-3 coexpression (Figure 3, B and C). We also confirmed that ColVI depletion did not affect KP tumor–derived cell proliferation (Supplemental Figure 5, A and B), consistent with the hypothesis that the dominant role of ColVI is specific to immunomodulation in the TME. To directly test the effect of COLVI on T cell–mediated killing, we employed the human TnMUC1-CART system introduced in Figure 1J (32). Longitudinal T cell–mediated cytolysis of STS-109 UPS cells expressing control or *COL6A1*-specific shRNAs revealed that COLVI depletion enhanced cytotoxic T cell function, phenocopying the effects of *YAP1* depletion (Figure 3, D and E, and Supplemental Figure 5C). To address the possibility that shCOL6A1 UPS cells (and shYAP1 cells in Figure 1J) are simply more susceptible than shScr cells to T cell–mediated apoptosis, we treated them with recombinant human TNF-α or IFN-γ, two cytolytic cytokines known to be produced by CD8^+^ T cells, and evaluated apoptosis by flow cytometry. We reasoned that equivalent doses of purified cytokines should elicit similar levels of apoptosis in shYAP1/shCOL6A1 cells and controls if CD8^+^ T cell function is truly enhanced in the setting of UPS cell–intrinsic *YAP1* or *COL6A1* deficiency. Purified cytokines did not increase shYAP1/shCOL6A1 UPS cell apoptosis relative to shScr controls, confirming enhanced CART cell cytotoxicity in the presence of reduced UPS cell–derived COLVI (Supplemental Figure 5, D and E). In fact, IFN-γ reduced late apoptosis in shYAP1 and shCOL6A1 UPS cells compared with controls, albeit modestly and inconsistently across cytokine concentrations and independent shRNAs. Consistent with these results, COLVI protein was detected extracellularly and in UPS cell culture–conditioned medium (Supplemental Figure 5, F–H), where it can suppress CART-TnMUC1–mediated cytolysis and promote T cell dysfunction.

In light of our in vitro findings that ColVI suppresses CD8^+^ T cell function, we investigated this relationship in vivo by generating control and *Col6a1* shRNA–expressing UPS tumors (syngeneic allograft of SKPY42.1 KP cells on a pure C57BL/6 background) in C57BL/6 hosts. In these immunocompetent mice, ColVI-deficient tumors were significantly smaller and slower growing than control tumors (Figure 3, F and G). We demonstrated that ColVI-dependent tumor growth is mediated by T cell inactivation by depleting CD8^+^ T cells in the syngeneic transplant system (Figure 3H and Supplemental Figure 5I), where control and shCol6a1 tumors grew at the same rate. We also generated syngeneic orthotopic tumors by injecting control and shCol6a1-expressing KP cells into the gastrocnemius muscles of immunocompetent C57BL/6 mice (Figure 3I and Supplemental Figure 5J). Flow cytometric analysis indicated that the proportion of CD8^+^ T cells expressing dysfunction markers, including Tox, Tim-3, CD39, and Lag3, was significantly decreased in ColVI-deficient tumors compared with controls (Figure 3, J and K). Together, these findings confirm that Yap1-mediated ColVI deposition in the UPS TME promotes CD8^+^ T cell dysfunction and immune evasion.

### ColVI colocalizes with and remodels ColI fibers in the UPS TME.

Next, we explored the mechanism by which COLVI promotes CD8^+^ T cell dysfunction in the UPS TME. We first asked whether CD8^+^ T cell dysfunction is induced following direct interaction with deposited COLVI via known COLVI receptors. To test this hypothesis, we developed a second in vitro system by incorporating purified human COLVI into COLI-containing hydrogels. COLI, a fibrillar collagen, is a widely used hydrogel scaffold because of its mechanical stability, hydrophilicity, versatility, in vivo abundance, and ease of extraction (47). COLVI, which forms microfilaments instead of fibers, does not possess all of these properties and thus cannot be used to generate hydrogels independently; moreover, unlike COLI, which can be found in isolation and need not interact with other matrix proteins in vivo, COLVI is always found bound to other ECM molecules and/or cell surface proteins (48). Activated human CD8^+^ T cells were then cultured on these COLVI-containing hydrogels, allowing us to assess the impact of purified matrix proteins on CD8^+^ T cells in a 3D environment. Subsequently, we blocked the known COLVI receptors ITGB1, NG2/CSPG4, CMG2/ANTXR2, and ITGAV (Supplemental Figure 6A). ITGAV and ITGB1 can bind multiple collagen species, including both COLVI and COLI; however, to our knowledge, NG2 and CMG2 are specific for COLVI (48–51). ITGB1 or NG2 neutralization with blocking antibodies did not affect CD8^+^ T cell dysfunction (TIM-3/PD-1 coexpression), nor did it restore CD8^+^ T cell proliferation (KI67 positivity). Similar results were obtained following treatment of human CD8^+^ T cells with cilengitide, a selective inhibitor of α_v_β_3_ and α_v_β_5_ integrins (52), and with activated CD8^+^ T cells from *Cmg2^–/–^* mice (53) (Supplemental Figure 6, B–F). These results suggest that CD8^+^ T cell dysfunction is not strongly modulated by canonical COLVI receptors. However, we cannot exclude the potential involvement of ITGAV and/or ITGB1, as neutralization of these receptors would likely block both T cell–COLVI and T cell–COLI interactions in our hydrogel system.

**Figure 4:**
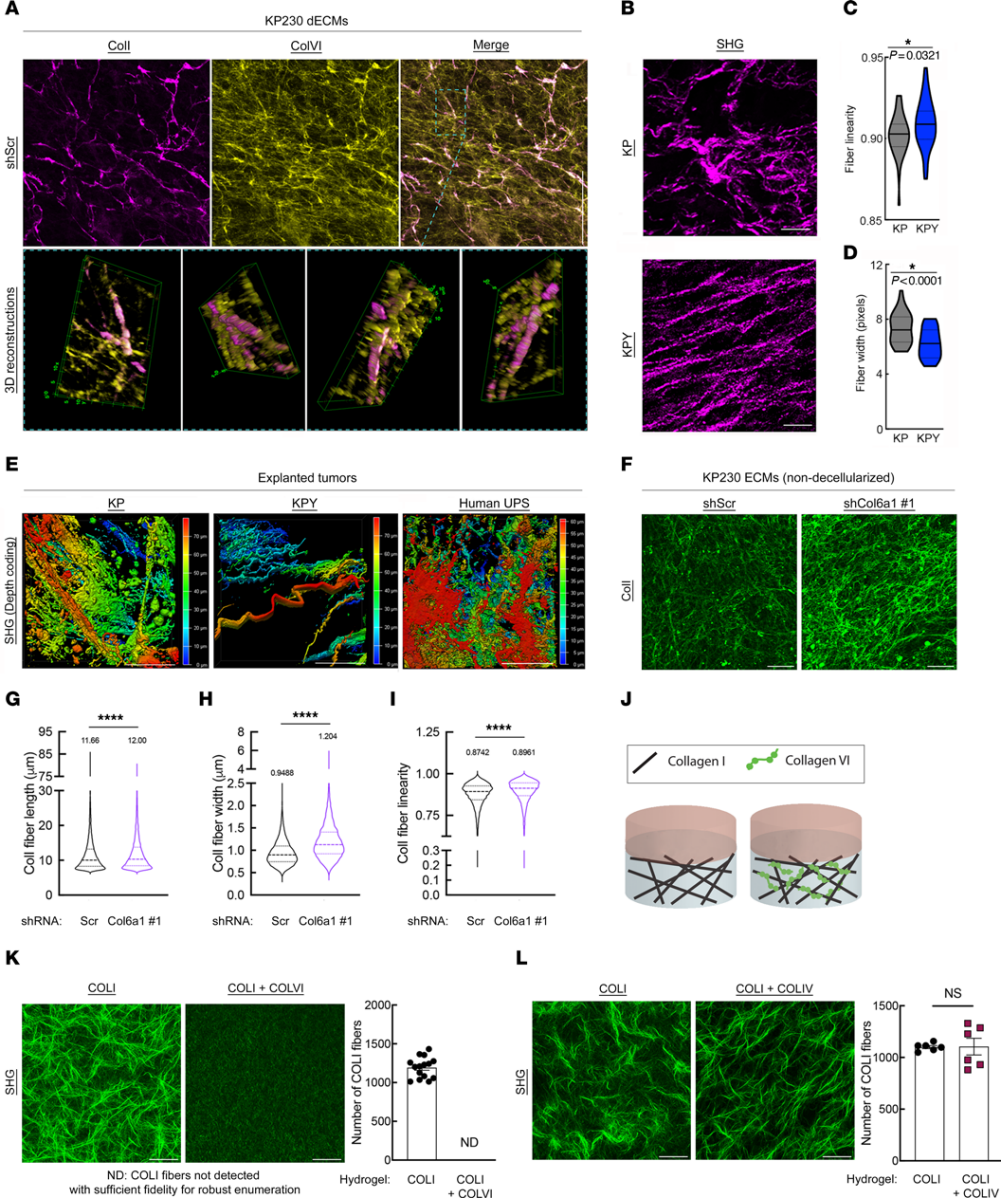
ColVI interacts with and remodels ColI in the UPS TME. (**A**) Representative confocal micrographs (maximum-intensity *Z*-projections) and 3D reconstructions showing ColI and ColVI coimmunofluorescence in KP cell–derived dECMs. Scale bar: 100 μm. (**B**) Representative multiphoton second-harmonic generation (SHG) images (maximum-intensity *Z*-projections) of KP and KPY tumor sections. Scale bars: 50 μm. (**C** and **D**) Violin plots of CT-FIRE analysis of images from **B**. Mean fiber width and linearity were plotted for at least 5 separate fields (*n* = 5 mice per genotype); 2-tailed unpaired *t* test. (**E**) Representative depth-coded SHG images of human UPS, KP, and KPY explanted live tumors. Red, SHG signal farthest from the objective/greatest relative tissue depth; blue, SHG signal closest to the objective/shallowest relative tissue depth. Scale bars: 50 μm. (**F**) Representative confocal micrographs (maximum-intensity *Z*-projections) of extracellular ColI immunofluorescence in ECMs (non-decellularized) generated from control and shCol6a1 KP cells. Scale bars: 50 μm. (**G**–**I**) Violin plots depicting CT-FIRE analysis of images in **F**. Fiber length (**G**), width (**H**), and linearity (**I**) were plotted from 7 independent fields across multiple dECMs per condition. Numbers above violin plots indicate means. Thick and thin dotted lines within the shapes denote medians and quartiles 1 and 3, respectively. (**J**) Schematic of in vitro hydrogel system to assess how purified COLVI impacts purified COLI structure/organization. (**K**) Representative SHG images (maximum-intensity *Z*-projections with ×2 optical zoom) and quantification of COLI fiber number in COLI-alone and COLI plus COLVI hydrogels. Scale bars: 50 μm. (**L**) Representative SHG images (maximum-intensity *Z*-projections with ×2 optical zoom) and quantification of COLI fiber number in COLI-alone and COLI plus COLIV hydrogels. Scale bars: 50 μm. For **K** and **L**, quantification was performed for at least 6 independent fields across multiple hydrogels per condition. Brightness and contrast of all micrographs in Figure 4 were adjusted for publication.

In the absence of a direct mechanism connecting ColVI receptors to T cell dysfunction, we investigated potential indirect mechanisms. ColVI binds to many ECM proteins, including ColI, one of the most abundant collagens in mammalian tissues (48, 54). In one study, COLI induced peripheral blood CD8^+^ T cell proliferation in vitro when used in conjunction with CD3/TCR stimulation (55). Therefore, we hypothesized that Yap1-mediated ColVI deposition promotes CD8^+^ T cell dysfunction by altering ColI content and/or organization in the UPS ECM. We first examined the effect of *Col6a1* depletion on ColI levels in KP cells in vitro, but generally observed no consistent changes in ColI gene or protein expression between *Col6a1*-deficient and control cells (Supplemental Figure 7, A–G). However, marked colocalization between ColI and ColVI in control (shScr) KP dECMs was observed, indicating a potential physical interaction between these 2 proteins (Figure 4A, Supplemental Figure 7H, and Supplemental Videos 1–3). Therefore, we considered the possibility that ColVI remodels ColI in the UPS TME, with potential implications for CD8^+^ T cell function.

To test this hypothesis, we began by examining the architecture of fibrillar collagen molecules in explanted GEMM tumors. Using multiphoton second-harmonic generation (SHG) imaging, we identified significant alterations to fibrillar collagen organization, including thinner and straighter fibers in KPY tumors compared with KP (Figure 4, B–D). Importantly, these changes in fibrillar collagen structure occurred despite similar levels of ColI gene expression and protein deposition in KP and KPY tumors (Figure 2A, Supplemental Figure 3D, and Supplemental Figure 8, A–C). Similar results were observed in SAHA/JQ1–treated tumors compared with controls (Supplemental Figure 8, D–F). We also found that the fibrillar collagen structure of explanted human UPS tumors recapitulated that of KP tumors, confirming that our GEMMs successfully reproduce this aspect of UPS biology (Figure 4E and Supplemental Videos 4–6). To explore the impact of ColVI on ColI organization more directly, we examined extracellular ColI immunofluorescent staining patterns in control and *Col6a1*-deficient KP cell–derived ECMs (Figure 4F). In this experiment, matrices were not decellularized to circumvent the potential (albeit minor) changes in ECM structure induced by decellularization. ColI fibers in shCol6a1 ECMs were significantly longer, straighter, and wider than those in shScr ECMs, with significantly different orientation distributions, confirming ColVI-mediated remodeling (Figure 4, G–I, and Supplemental Figure 8G). Finally, we asked whether COLVI alters COLI structure directly or indirectly through other mechanisms by performing SHG imaging of hydrogels containing purified COLI alone, or COLI together with purified COLVI (Figure 4J). As SHG only detects fibrillar collagen molecules, COLI, but not COLVI, is imaged in this assay. Remarkably, the addition of COLVI (250 μg/mL) to our hydrogel system nearly abolished the formation of COLI fibers and higher-level structures (e.g., fiber bundles; Figure 4K). In contrast, COLI fibers remained abundant in the presence of a different non-fibrillar collagen, collagen type IV (COLIV; also 250 μg/mL), underscoring the potential specificity of the COLI-COLVI relationship (Figure 4L). Therefore, we conclude that ColVI directly modifies ColI fiber architecture in the UPS TME.

### ColI opposes ColVI and abrogates CD8^+^ T cell dysfunction.

**Figure 5:**
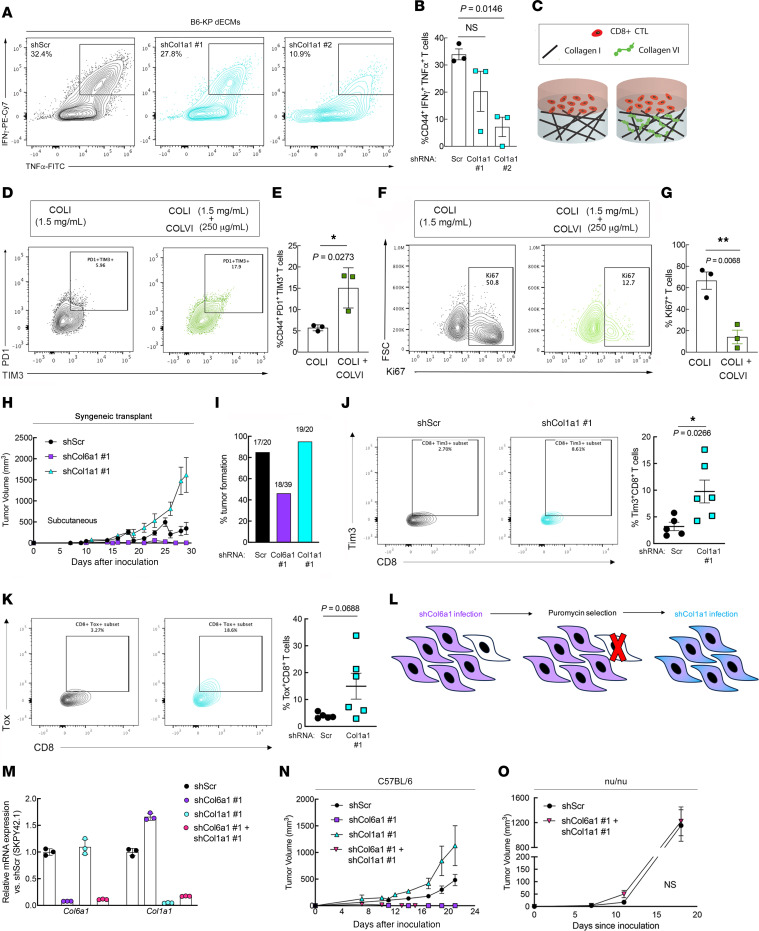
ColVI-mediated CD8^+^ T cell dysfunction is restored in the presence of ColI. (**A** and **B**) Representative flow cytometry plots (**A**) and quantification (**B**) of IFN-γ and TNF-α coexpression in CD44^+^CD8^+^ T cells incubated on dECMs from control or shCol1a1 KP cells. Each point represents T cells from an individual mouse; 1-way ANOVA with Dunnett’s test vs. shScr. The shScr plot and data are identical to those in Figure 2J (performed in the same experiment). (**C**) Schematic of in vitro hydrogel system to test how ColI impacts ColVI-mediated CD8^+^ T cell dysfunction. CTL, cytotoxic T lymphocyte. (**D** and **E**) Representative flow cytometry plots (**D**) and quantification (**E**) of activated human CD8^+^CD44^+^ T cells showing TIM-3 and PD-1 coexpression after incubation on hydrogels containing purified COLI with or without purified COLVI; 2-tailed unpaired *t* test. (**F** and **G**) Representative flow cytometry plots (**F**) and quantification (**G**) of Ki67 in activated human CD8^+^CD44^+^ T cells cultured as in **D** and **E**; 2-tailed unpaired *t* test. (**H**) Tumor growth curves from subcutaneous (flank) syngeneic transplant of 5 × 10^5^ KP cells (SKPY42.1 cell line) expressing control, *Col6a1*, or *Col1a1*-targeting shRNAs in C57BL/6 mice. Data are from 2 independent experiments (total *n* = 20 for shScr and shCol1a1; *n* = 39 for shCol6a1). (**I**) Tumor formation rates from **H**. (**J** and **K**) Representative contour plots and quantification of Tim-3 (**J**) and Tox (**K**) expression in CD8^+^ T cells in tumors from **H**. Each point represents an individual tumor; 2-tailed unpaired *t* test with Welch’s correction. (**L**) Schematic depicting strategy for depleting *Col6a1* and *Col1a1* in the same UPS cell population. (**M**) Validation of *Col6a1* and *Col1a1* expression in UPS cells from **L** (SKPY42.1 cell line). Technical replicates. (**N** and **O**) Tumor growth curves from subcutaneous syngeneic transplant of 1 × 10^6^ KP cells (SKPY42.1 cell line) from **M** in C57BL/6 (**N**) and nu/nu (**O**) mice.

We next sought to understand mechanistically how ColI-ColVI interactions impact CD8^+^ T cells, hypothesizing that ColVI triggers dysfunction indirectly by remodeling ColI in the TME. To test the impact of ColI on CD8^+^ T cell function, we incubated activated murine CD8^+^ T cells on dECMs from control or *Col1a1*-deficient B6-KP cells. Unlike in the setting of *Yap1* and *Col6a1* deficiency (Figures 2 and 3), the proportion of IFN-γ^+^TNF-α^+^ CD8^+^ T cells was reduced following culture on *Col1a1-*deficient dECMs, indicating decreased cytolytic capacity (Figure 5, A and B). We then cultured activated human CD8^+^ T cells on hydrogels containing purified COLI alone, or COLI together with purified COLVI (Figure 5C). The proportion of PD-1/TIM-3–coexpressing CD8^+^ T cells was significantly reduced on COLI gels versus COLVI-containing gels (Figure 5, D and E); TIM-3 median fluorescence intensity was similarly decreased (Supplemental Figure 9A). Additionally, CD8^+^ T cell proliferative capacity (KI67 positivity) was improved on COLI gels relative to COLI + COLVI gels (Figure 5, F and G). The proportion of cytolytic IFN-γ^+^TNF-α^+^ CD8^+^ T cells was also modestly elevated in the presence of COLI alone (Supplemental Figure 9B). We then tested the specificity of the effect of COLI-COLVI interactions on CD8^+^ T cell function by substituting COLIV for COLVI in this assay. Remarkably, CD8^+^ T cell dysfunction was not significantly impacted by the addition of COLIV to COLI-containing hydrogels (Supplemental Figure 9C), consistent with our SHG data (Figure 4L), and further illustrating the specificity of the COLI-COLVI relationship.

The stiff, fibrotic microenvironments in desmoplastic solid tumors are well known to activate Yap1 (27). Therefore, we ascertained whether COLVI drives CD8^+^ T cell dysfunction by increasing ECM stiffness and potentiating Yap1 signaling. We queried Yap1 expression and subcellular localization in control, shCol6a1, and shCol1a1 KP cells, but did not detect significant differences in gene expression, protein levels, or S127 phosphorylation, an established surrogate for cytoplasmic retention and degradation (Supplemental Figure 9, D–F). Consistent with this observation, hydrogel stiffness was not significantly altered by the addition of COLVI (Supplemental Figure 9G).

We also investigated the involvement of a ColI receptor, Lair1, in CD8^+^ T cell dysfunction, given a recent report that Lair1 negatively regulates CD8^+^ T cell activity and may promote immunotherapy resistance in lung cancer (56). Analysis of publicly available single-cell RNA sequencing data from KP UPS tumors (GSE144507; ref. 57) demonstrated that *Lair1* was expressed predominantly on tumor-associated macrophages and minimally on CD8^+^ T cells (Supplemental Figure 9H). Moreover, *LAIR1* (but not *COL1A1* itself) was modestly associated with improved overall UPS patient survival (Supplemental Figure 9, I and J), inconsistent with its putative role promoting CD8^+^ T cell immunosuppression (56). These results argue against the involvement of *Lair1* in UPS matrix-mediated immune evasion. Finally, we confirmed that neither molecular diffusion rates throughout, nor oxygen concentrations within, the hydrogel system were substantially impacted by the addition of COLVI, demonstrating that COLVI-induced CD8^+^ T cell dysfunction in this model is not likely triggered by differential nutrient and/or oxygen availability (Supplemental Figure 9, K and L). Together, these observations clearly indicate that COLI abrogates COLVI-mediated CD8^+^ T cell dysfunction, and demonstrate the relative importance of ECM signaling over Yap1 hyperactivation in this process.

Our findings thus far suggested that ColVI restrains ColI-mediated CD8^+^ T cell activity and proliferation. To test this hypothesis in vivo, we generated subcutaneous syngeneic tumors by injecting SKPY42.1 cells expressing control, *Col1a1*-targeting, or *Col6a1*-targeting shRNAs into C57BL/6 mice (Figure 5H). In this immunocompetent setting, *Col1a1*-deficient tumors grew more rapidly than both control and *Col6a1*-deficient tumors. Additionally, shCol1a1 and shScr tumors developed with similar efficiency (85% vs. 95%), whereas shCol6a1 tumors only formed in 46.2% of mice (Figure 5I). Of the shCol6a1 tumors that did form, 83.3% rapidly regressed before they reached 100 mm^3^. Similar results were obtained in immunocompetent syngeneic orthotopic tumor models (Supplemental Figure 10, A and B). Importantly, shCol1a1 tumor–bearing mice experienced worse survival than mice bearing control tumors, whereas survival of shCol6a1 tumor–bearing mice was improved (Supplemental Figure 10C). Furthermore, Tim-3 and Tox were upregulated on CD8^+^ T cells in *Col1a1*-deficient UPS tumors compared with controls (Figure 5, J and K), indicating increased dysfunction due to ColI loss. Impressively, when we assessed the impact of *Col1a1* depletion on KP cell growth in vitro, *Col1a1*-deficient cells proliferated more slowly than controls, reflecting a discrepancy between ColI function in vitro and in vivo (Supplemental Figure 10D). These results confirm that ColI in the UPS ECM controls tumor growth by enabling host antitumor immunity, whereas the aberrant deposition of ColVI opposes ColI and promotes immune evasion.

To ascertain whether ColVI or ColI plays the dominant role in matrix-mediated CD8^+^ T cell dysfunction, we depleted *Col6a1* and *Col1a1* in the same population of SKPY42.1 cells and injected them into recipient syngeneic C57BL/6 mice (Figure 5, L and M). Cells deficient for both collagens formed tumors at intermediate rates between those deficient for Col6a1 or Col1a1 individually (Supplemental Figure 10E), but the resulting tumors (herein referred to as “double-knockdown tumors”) rapidly regressed before they reached approximately 100 mm^3^ (Figure 5N and Supplemental Figure 10F), phenocopying shCol6a1 tumors. To confirm T cell–dependent regression, we generated control and double-knockdown tumors in nu/nu mice, in which mature T cells are lacking but innate immune cells are present. In this critical experiment, no significant differences in tumor formation or growth were observed (Figure 5O and Supplemental Figure 10, G and H), confirming T cell–mediated double-knockdown tumor regression in the syngeneic model (Figure 5N). Thus, we conclude that ColVI is dominant over ColI in UPS matrix-mediated immune evasion.

### ColVI promotes T cell dysfunction by disrupting CD8^+^ T cell autophagic flux.

**Figure 6:**
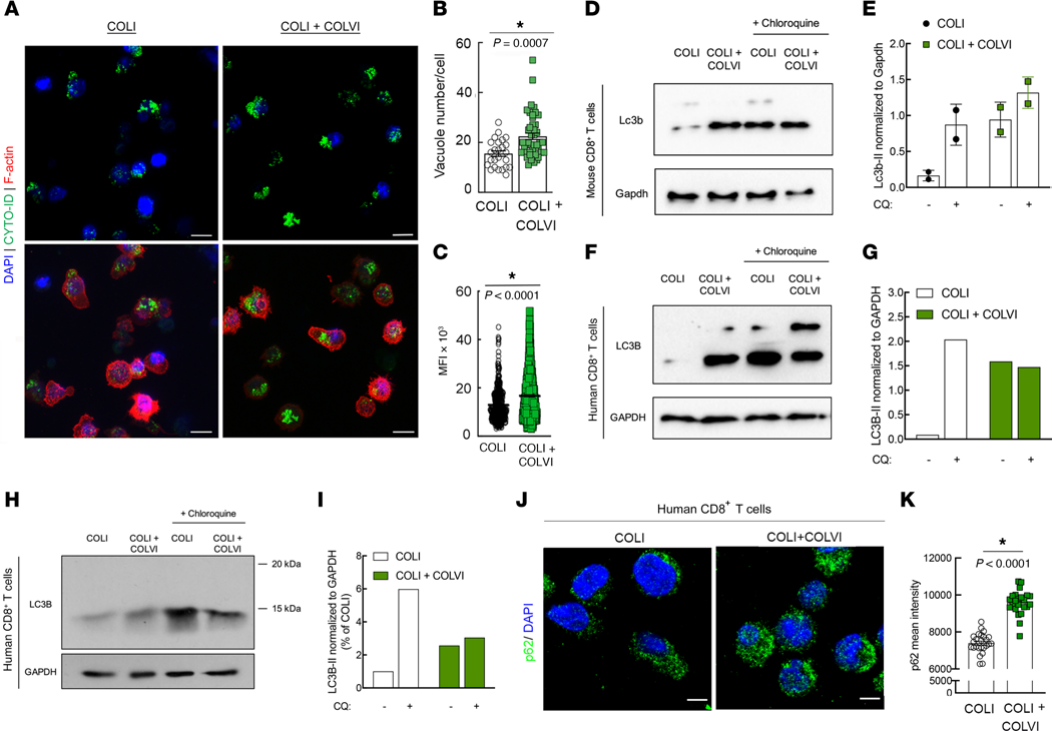
ColVI disrupts CD8^+^ T cell autophagic flux. (**A**–**C**) Visualization (**A**) and quantification (**B** and **C**) of autophagosomes in CD8^+^ human T cells cultured on hydrogels containing purified COLI with or without purified COLVI; 2-tailed unpaired *t* test. (**D** and **E**) Western blot (**D**) and quantification (**E**) of Lc3b-II expression in murine CD8^+^ T cells cultured on purified COLI-containing hydrogels in the presence or absence of purified COLVI, with or without chloroquine (CQ) treatment; 2-tailed unpaired *t* test; *n* = 2; SD. (**F** and **G**) Western blot (**F**) and quantification (**G**) of LC3B-II expression in human CD8^+^ T cells cultured on purified COLI-containing hydrogels in the presence or absence of purified COLVI, with or without CQ treatment. (**H** and **I**) Western blot (**H**) and quantification (**I**) of LC3B-II expression in human CD8^+^ T cells cultured on purified COLI-containing hydrogels in the presence or absence of purified COLVI, with or without CQ treatment. Molecular weight marker positions are shown to demonstrate that the single LC3B band detected in this experiment corresponds to the reported molecular weight for LC3B-II (14–16 kDa for LC3B-II vs. 16–18 kDa for LC3B-I). Samples from **F**–**I** were generated from cells from different donors, and are shown separately because the analyses were conducted at different institutions using different detection methods (digital fluorescent detection vs. chemiluminescence on film). (**J** and **K**) Representative images (**J**) and quantification (**K**) of p62 immunofluorescence in human CD8^+^ T cells cultured on purified COLI-containing hydrogels with or without purified COLVI; 2-tailed unpaired *t* test. Scale bars: 10 μm (A); 15 μm (J).

To date, an immunosuppressive role of ColVI has not been documented in tumors. We therefore sought to identify the downstream mechanism by which ColVI deposition causes T cell dysfunction, and were intrigued by the reported ability of ColVI to modulate autophagy in fibroblasts and muscle tissue (58). Indeed, autophagy is a central regulator of T cell metabolism and is essential for T cell activation (59). To assess whether ColVI impacted CD8^+^ T cell autophagy, we encapsulated T cells in purified COLVI-containing hydrogels and visualized autophagosomes in situ. T cell autophagosomes were brighter and more numerous in the presence of COLVI versus COLI alone (Figure 6, A–C). To determine whether COLVI caused autophagosome accumulation by inducing autophagy or by disrupting autophagic flux and autophagosome clearance, we treated activated CD8^+^ T cells with chloroquine (CQ) in the presence or absence of COLVI and evaluated LC3B-II expression. In murine T cells, inhibiting autophagic flux with CQ did not further increase Lc3b-II, indicating that COLVI disrupts autophagic flux but does not induce autophagy (Figure 6, D and E). Activated human peripheral blood CD8^+^ T cells generally showed the same trend (Figure 6, F–I). We confirmed this result by staining for p62, a protein rapidly degraded during autophagy induction. In the presence of COLVI, p62 accumulated within T cells as evidenced by increased mean p62 signal intensity (Figure 6, J and K). Together, these results indicate that extracellular COLVI inhibits CD8^+^ T cell autophagic flux.

To explore the broader impacts of our findings, we next considered whether COLVI might also impact CD8^+^ T cell function in other cancer types. Analysis of TCGA PanCancer RNA sequencing data revealed that, like in sarcomas, *COL6A1*, *COL6A2*, and *COL6A3* are highly expressed in pancreatic ductal adenocarcinoma (PDAC) (Supplemental Figure 11A). Indeed, PDAC tumors contain abundant desmoplastic stroma, secreted primarily by cancer-associated fibroblasts (CAFs) in the TME (14). Therefore, we generated dECMs from PDAC-CAFs isolated from 3 independent human tumors and confirmed their ability to secrete COLVI (Supplemental Figure 11B). We then generated dECMs from control and shCOL6A1-expressing PDAC-CAFs and incubated them with activated human CD8^+^ T cells. Surprisingly, the proportion of PD-1/TIM-3–coexpressing CD8^+^ T cells was not altered by exposure to *COL6A1*-deficient versus -replete PDAC-CAF–derived matrix (Supplemental Figure 11, C and D). Thus, unlike in UPS, COLVI does not appear to modulate CD8^+^ T cell function in PDAC. Given a recent report that fibroblast-derived ColI (containing Col1a1/Col1a2 heterotrimers) is distinct from PDAC cancer cell–derived ColI (containing Col1a1 homotrimers that possess oncogenic properties) (60), we then asked whether the differential immunomodulatory capacity of CAF-derived versus UPS cell–derived ECM resulted from the production of heterotrimeric versus homotrimeric ColI, respectively. However, like fibroblasts (and unlike PDAC cancer cells) (60), *Col6a1*-sufficient and -deficient UPS cells secreted both Col1a1 and Col1a2 (Supplemental Figure 11E), demonstrating that differential ColI trimer composition likely does not underlie the divergent effects of CAF-derived versus UPS cell–derived matrix on CD8^+^ T cell function. Taken together, these data underscore critical differences in matrix protein composition between sarcomas and carcinomas, and highlight the potential specificity of the ColVI–CD8^+^ T cell relationship to mesenchymal tumors.

### COLVI as a potential prognostic and diagnostic biomarker in human STS.

**Figure 7:**
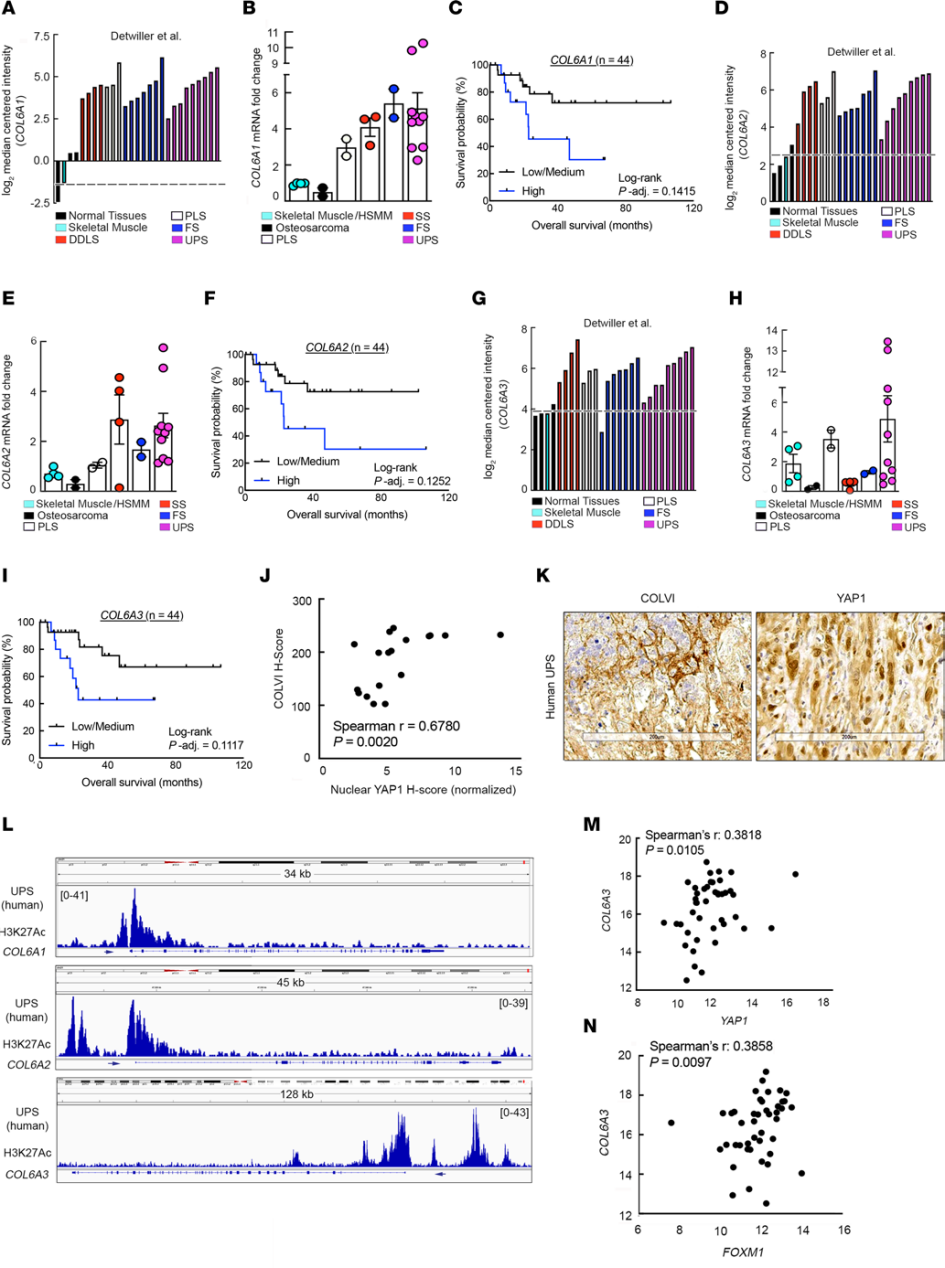
YAP1 and COLVI expression/activity are correlated in human UPS tumors. (**A**) *COL6A1* gene expression levels in specimens from the Detwiller et al. sarcoma data set (Oncomine) (61). DDLS, dedifferentiated liposarcoma; PLS, pleomorphic liposarcoma; FS, fibrosarcoma. (**B**) qRT-PCR analysis of *COL6A1* expression in human sarcoma and normal skeletal muscle tissue specimens (Hospital of the University of Pennsylvania [HUP]). SS, synovial sarcoma. (**C**) Kaplan-Meier overall survival curves of UPS patients in TCGA-SARC stratified by intratumoral *COL6A1* gene expression levels. (**D**) *COL6A2* gene expression levels in specimens from the Detwiller et al. sarcoma data set. (**E**) qRT-PCR analysis of *COL6A2* expression in human sarcoma and normal skeletal muscle tissue specimens (HUP). (**F**) Kaplan-Meier overall survival curves of UPS patients in TCGA-SARC stratified by intratumoral *COL6A2* gene expression levels. (**G**) *COL6A3* gene expression levels in specimens from the Detwiller et al. sarcoma data set. (**H**) qRT-PCR analysis of *COL6A3* expression in human sarcoma and normal skeletal muscle tissue specimens (HUP). (**I**) Kaplan-Meier overall survival curves of UPS patients in TCGA-SARC data set stratified by intratumoral *COL6A3* gene expression levels. For **C**, **F**, and **I**, tertiles (low, medium, high) represent one-third of patients. (**J**) Correlation of COLVI and nuclear YAP1 immunostaining in UPS tumor specimens (HUP). Each point represents an individual specimen. (**K**) Representative IHC images from **J**. Scale bars: 200 μm. (**L**) Publicly available ChIP-Seq data (GSE97295) of *COL6A1*, *COL6A2*, and *COL6A3* promoter H3K27 acetylation in human UPS samples (HUP). (**M** and **N**) Correlation of *YAP1* with *COL6A3* (**M**) and *FOXM1* (**N**) gene expression in UPS tumors from TCGA-SARC.

To evaluate our experimental findings in a clinical context, we used data from multiple human UPS patient cohorts: the Detwiller et al. data set (61), TCGA-SARC, and surgical specimens from the Hospital of the University of Pennsylvania (HUP). Like *YAP1* (22), *COL6A1*, *COL6A2*, and *COL6A3* were upregulated in human UPS relative to normal muscle tissue and correlated with poor patient outcomes (Figure 7, A–I). Additionally, consistent with our in vitro and GEMM data demonstrating that Yap1 promotes ColVI deposition in the UPS TME, COLVI expression highly correlated with nuclear YAP1 staining, a surrogate for YAP1 transcriptional activity (Figure 7, J and K). Moreover, the *COL6A1*, *COL6A2*, and *COL6A3* promoters appeared transcriptionally active in these specimens given the presence of H3K27Ac marks at these loci (Figure 7L). Finally, *COL6A3* gene expression positively tracked with that of *YAP1* and the *YAP1* target gene *FOXM1* (Figure 7, M and N).

**Figure 8:**
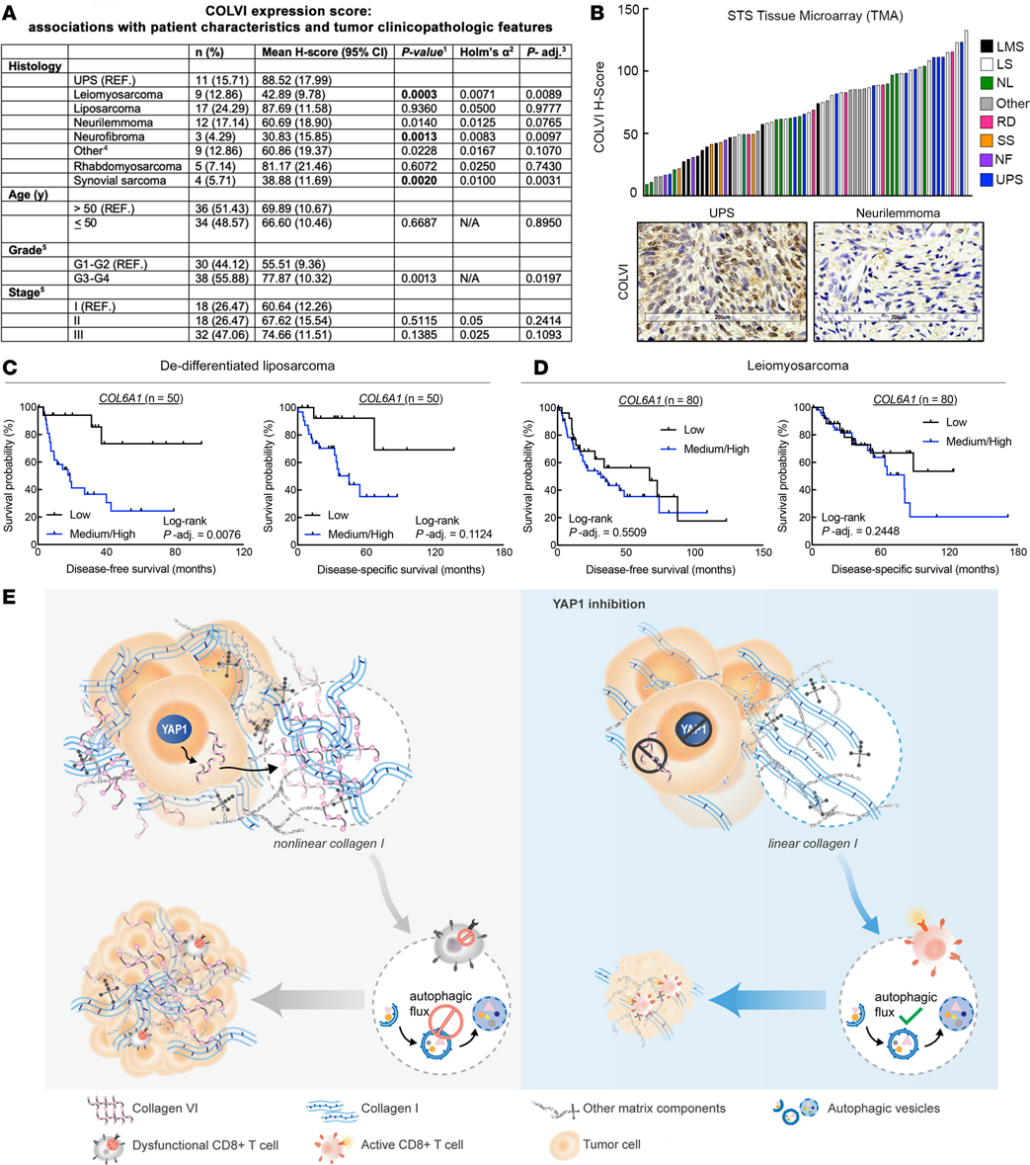
COLVI expression in the microenvironments of UPS and other soft-tissue sarcoma subtypes. (**A**) Association of IHC-based COLVI expression score with tumor subtype and clinicopathologic features. Sarcoma tissue microarray. ^1^Univariate linear models. ^2^In univariate analyses, the Holm-Bonferroni adjustment for multiple comparisons was performed for demographic or clinicopathologic variables with more than 2 levels, with α = 0.05. Results are considered statistically significant (bold text) if the univariate *P* value is smaller than the corresponding Holm’s α. ^3^Fully adjusted model (age, grade, stage, and histology). Correction for multiple comparisons was not performed owing to insufficient statistical power. ^4^Includes 2 alveolar soft-part sarcomas, 1 epithelioid hemangioendothelioma, 1 fibroma, 1 glomus tumor, 1 hemangioendothelial sarcoma, 1 hemangiopericytoma, 1 osteosarcoma, and 1 tenosynovial giant cell tumor. ^5^Excludes 2 benign cases. (**B**) Waterfall plot depicting IHC-based COLVI expression scores in individual tumors from **A**. LMS, leiomyosarcoma; LS, liposarcoma; NL, neurilemmoma; RD, rhabdomyosarcoma; SS, synovial sarcoma; NF, neurofibroma; STS, soft-tissue sarcoma. “Other” as described in **A**. Representative images of UPS and neurilemmoma are also shown. Scale bars: 200 μm. (**C** and **D**) Kaplan-Meier disease-free and disease-specific survival curves of dedifferentiated liposarcoma (**C**) and leiomyosarcoma (**D**) patients in TCGA-SARC stratified by *COL6A1* gene expression. Tertiles (low, medium, high) represent one-third of patients. (**E**) Model depicting study findings.

Finally, we explored the relationship between COLVI expression in UPS and other sarcoma subtypes using a tissue microarray (TMA). The mean UPS COLVI H-score (88.52; range, 105.73) was higher than those of all other subtypes, and significantly greater than those of leiomyosarcomas, neurofibromas, and synovial sarcomas (Figure 8, A and B). Importantly, the dynamic range of COLVI staining in the UPS TMA cohort was similar to that in the HUP cohort (Figure 7, J and K; range, 142.94). As UPS is more prevalent among older adults and presents with aggressive clinical features (18), we adjusted associations between COLVI H-score and histology for patient age, tumor grade, and tumor stage (Figure 8A). After controlling for these variables, relationships between COLVI H-score and histologic subtype were attenuated but remained statistically significant. Tumor grade was also significantly associated with COLVI H-score in univariate models. Furthermore, in TCGA-SARC, *COL6A1* gene expression was associated with reduced long-term survival among liposarcoma patients, where tumor COLVI expression levels are similar to those in UPS, but not among leiomyosarcoma patients, where tumor COLVI levels are significantly lower (Figure 8, C and D). Thus, COLVI expression may be a biomarker of clinical outcomes and immunotherapy sensitivity in some human sarcoma patients.

## Discussion

Until now, our understanding of the role of the ECM in antitumor immunity was primarily limited to its effects on leukocyte migration. Additionally, upstream mediators of aberrant ECM protein composition in the TME were poorly defined. Herein, we establish a more specific and mechanistic understanding of individual collagen molecules in the solid tumor ECM and their role in adaptive immunity (Figure 8E). We discovered that the highly expressed transcriptional coregulator Yap1 promotes the deposition of a pro-tumor matrix protein, ColVI, in the UPS ECM. In turn, ColVI opposes anti-neoplastic ColI molecules in the TME, altering their organization/architecture, and disrupts CD8^+^ T cell autophagic flux. Ultimately, this cascade facilitates CD8^+^ T cell dysfunction by upregulating inhibitory receptors, suppressing proliferation, and reducing effector function. Together, our findings describe a non-canonical role of Yap1 in the TME and establish a direct mechanistic link between specific ECM constituents and modulation of immune cell function.

Despite the incredible diversity and abundance of collagen superfamily molecules in solid tumors (12, 17), the effects of specific collagens and other matrix proteins on T cell effector function in these contexts are only beginning to be characterized. For example, extracellular collagen molecules induced CD8^+^ T cell exhaustion and attenuated responses to anti-Pd1 checkpoint therapy in murine lung cancer models (56). These phenotypes were reversed following inhibition of LOXL2, a collagen cross-linking enzyme, but were not attributed to a specific collagen type, potentially because LOXL2 inhibition disrupts multiple collagen species. Additionally, laminin-111 has been implicated in the inhibition of CD8^+^ T cell expansion and function in vitro; however, in vivo validation of these results has not been pursued (62). The authors did show that Matrigel, the primary component of which is laminin-111, may accelerate syngeneic mammary tumor growth in immunocompetent mice, but did not address whether Matrigel directly stimulates cancer cell proliferation in vivo (62). Furthermore, Robertson et al. (63) showed that COLIV may reduce T cell–mediated mammary carcinoma cell clearance, but did not establish a direct mechanistic link between COLIV and suppression of T cell function. Instead, the authors suggested that COLIV may induce a more immunosuppressive transcriptional/secretory profile in mammary carcinoma cells. However, because syngeneic mammary carcinoma cells and splenocytes were not used in that study (63), those data are challenging to interpret. Conversely, herein, we uncovered specific immunomodulatory roles of 2 distinct collagen species in UPS and directly linked aberrant ECM composition/organization to induction of CD8^+^ T cell dysfunction. Using multiple orthogonal in vitro and in vivo readouts, we discovered that ColVI and ColI possess opposing roles in this context, promoting and opposing immune evasion, respectively. The ColVI-mediated dysfunction program observed herein upregulated multiple T cell inhibitory receptors and dysfunction markers, suppressed CD8^+^ T cell proliferation, and blunted CD8^+^ T cell cytolytic capacity. In contrast, ColI was a tumor suppressor in vivo and reduced CD8^+^ T cell dysfunction relative to ColVI. These observations are consistent with recent studies demonstrating the stimulatory effects of COLI on CD8^+^ T cell function. For example, COLI costimulation enhanced peripheral blood–derived effector T cell expansion in vitro (55), and increased intratumoral T cell content and activation gene expression in PDAC models in vivo (15). In contrast, a study of 3D culture models reported that high COLI density suppressed T cell proliferation, cytolytic marker gene expression, and cytolytic function (64). Taken together, these studies suggest that the effects of ColI on CD8^+^ T cells are complex and potentially context specific. Nevertheless, our study clearly shows that ColI is required for CD8^+^ T cell function in UPS. By extension, stromal depletion strategies that reduce COLI deposition in the UPS TME could elicit detrimental outcomes.

One of the most intriguing findings from our study is that ColVI in the UPS TME directly remodels extracellular ColI. We suspect that this ColVI-mediated matrix remodeling masks binding motifs on ColI, such as RGD (Arg-Gly-Asp) sites or GXXGER consensus sequences, that would otherwise facilitate tumoricidal ColI–CD8^+^ T cell interactions. However, the identity of the receptor on CD8^+^ T cells mediating interactions with ColI in the UPS TME remains an open question. We excluded the possibility that Lair1 may be involved because it was not expressed on CD8^+^ T cells in KP tumors. Similarly, the involvement of another ColI receptor, Ddr1, is unlikely, given Ddr1’s previously reported role in the negative regulation of CD8^+^ T cell migration/infiltration in carcinomas (65). Conversely, certain ColI-binding integrins such as Itga1, Itgav, and Itgb1 may be candidates given their putative roles promoting CD8^+^ T activity (66–68), but are challenging to study in UPS because they bind to both ColI and ColVI (48–51). Therefore, careful biochemical studies are required to fully elucidate the mechanism by which ColI promotes CD8^+^ T cell activity and inhibits immune evasion in UPS.

Senescence, functional exhaustion, insufficient homeostatic proliferation, deletion, and altered metabolism are all largely T cell–intrinsic mechanisms that can hamper endogenous and engineered T cell–mediated antitumor immunity (1). Our findings offer an alternative model in which cancer cell–intrinsic biology drives failure of cytotoxic T cell activity by indirectly interfering with T cell autophagic flux. Autophagy is rapidly induced upon T cell activation, and the essential autophagy genes *Atg5* and *Atg7* are critical for mature T cell survival, activation, and expansion (69, 70). Moreover, disrupting T cell autophagic flux hinders clearance of damaged mitochondria, resulting in increased reactive oxygen species (ROS) generation and T cell apoptosis (70). Thus, whether and how aberrant ColVI deposition influences ROS production in T cells with dysregulated autophagic flux is an important direction for future research.

Our study has multiple implications for the clinical management of human UPS. First, as UPS is a diagnosis of exclusion, some pleomorphic neoplasms classifiefd as “UPS” are more likely to be other high-grade sarcomas or pseudosarcomas (71). Thus, given the significantly increased COLVI levels in UPS relative to several other STS subtypes, COLVI may be a useful diagnostic biomarker for distinguishing UPS from other dedifferentiated pleomorphic tumors. Second, we found that Pd1 blockade extended survival of KPY mice, but not KP. Therefore, anti-Pd1 treatment was insufficient to reinvigorate dysfunctional effector T cells in KP mice, but did preserve CD8^+^ T cells with robust cytolytic function in KPY. Finally, our findings show that Yap1 facilitates immune evasion by modulating TME composition and organization, and indicate that individual collagens may have unique or opposing effects on UPS patient responses to T cell–based therapies. Specifically, COLVI in the UPS ECM may suppress responses to immune checkpoint blockade, whereas COLI may potentiate efficacy. Thus, our study underscores the critical need to systematically evaluate the roles of individual ECM components in immunoregulation. Furthermore, our data specifically implicate YAP1 and/or COLVI targeting as promising strategies by which to improve the efficacy of checkpoint blockade and other T cell–based therapies in UPS, and potentially other desmoplastic solid tumors.

## Methods

Detailed methods are provided in the Supplemental Methods and Supplemental Histopathology Appendix.

### Sex as a biological variable.

For GEMM studies, similar findings were observed in both sexes, so results are reported for both together. Syngeneic transplant studies were performed in all females, but the results are also expected to be relevant to males. Data from TCGA-SARC and the sarcoma TMA are reported for both sexes together. HUP human specimens were completely deidentified.

### Statistics.

Analyses were performed using GraphPad Prism. Presentation of biological versus technical replicates is indicated in the legends. Unless otherwise specified, in vitro experiments were replicated at least 3 times, and data show mean ± SEM. Statistics are not shown for experiments with *n* less than 3. Unpaired 2-tailed *t* tests and 1-way ANOVAs were used to compare 2 or 3 group means, respectively. Two-way repeated-measures ANOVAs, mixed models, or nonlinear regressions were used for in vivo tumor measurements. For correlations, Spearman’s coefficient was used if at least 1 data set was not normally distributed. Pearson’s coefficient was used if both data sets were normally distributed. Shapiro-Wilk test was used to assess normality. *P* less than 0.05 was considered statistically significant. For Kaplan-Meier analyses of TCGA-SARC data, we preselected 4 gene expression cutoffs to identify the “optimal” cutoff for detecting gene-survival associations (72). Identical cutoffs were used in all analyses to avoid bias. The log-rank *P* values from each comparison were then adjusted for multiple comparisons using the 2-stage linear step-up procedure of Benjamini, Krieger, and Yekutieli (false discovery rate, 5%). All comparisons performed for each gene/survival endpoint combination and their associated adjusted *P* values are shown in Supplemental Table 1.

### Study approval.

All experiments were performed in accordance with NIH guidelines and approved by the UPenn Institutional Animal Care and Use Committee (approval 805758). Studies with human specimens were not considered human-subjects research (samples were deidentified and not collected exclusively for research).

### Data availability.

A Supporting Data Values file is available in accordance with *JCI* policy. Additional data and materials are available upon reasonable request.

## Author contributions

AMF and HCP contributed equally. AMF’s name is listed first because she coordinated all revision efforts with the *JCI* while HCP pursued other professional opportunities. TSKEM, SG, JAF, and MH conceptualized the study. AMF, HCP, YL, and JAF established methodology. AMF, HCP, YL, VMIN, HS, AD, RSK, GEC, EFW, IM, and NS performed validation. AMF, HCP, YL, RSK, SD, and GEC performed formal analysis. HCP, AMF, YL, VMIN, HP, HS, AD, RSK, SD, GEC, EFW, IM, DN, and NS performed investigation. AMF, YL, and MVG performed data curation. HH, DJZ, JGT, AW, KW, MH, JAF, SG, and TSKEM provided resources. AMF, HCP, and TSKEM drafted the manuscript. AMF, HCP, and TSKEM reviewed and edited the manuscript. AMF, HCP, YL, EFW, JAF, and TSKEM performed visualization. MH, JAF, SG, and TSKEM supervised the study. TSKEM performed project administration. SG and TSKEM acquired funding.

## Supplementary Material

Supplemental data

Unedited blot and gel images

Supplemental video 1

Supplemental video 2

Supplemental video 3

Supplemental video 4

Supplemental video 5

Supplemental video 6

Supporting data values

## Version 1 04/23/2024

In-Press Preview

## Version 2 06/03/2024

Electronic publication
