# Supplementary material for: Oncogene-induced matrix reorganization controls CD8+ T cell function in the soft-tissue sarcoma microenvironment
Source: J Clin Invest. 2024 Apr 23;134(11):e167826. doi: 10.1172/JCI167826 (PMC11142734; doi:10.1172/JCI167826)
Supplement: Unedited blot and gel images [file jci-134-167826-s045.pdf]

kras

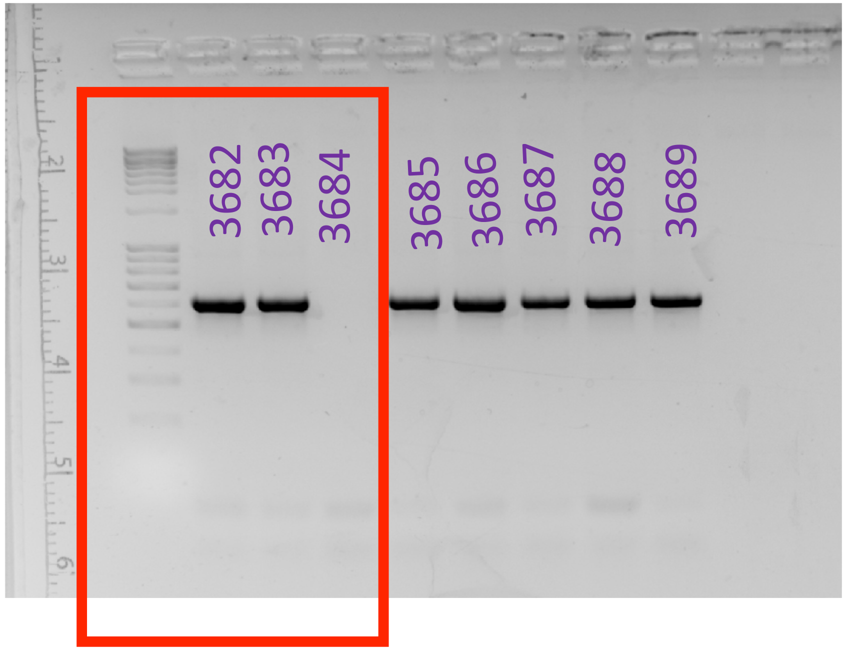

**KrasG12D bands were used in Fig. 1B

All are KrasG12G/+ except 3684 which are wt +/+

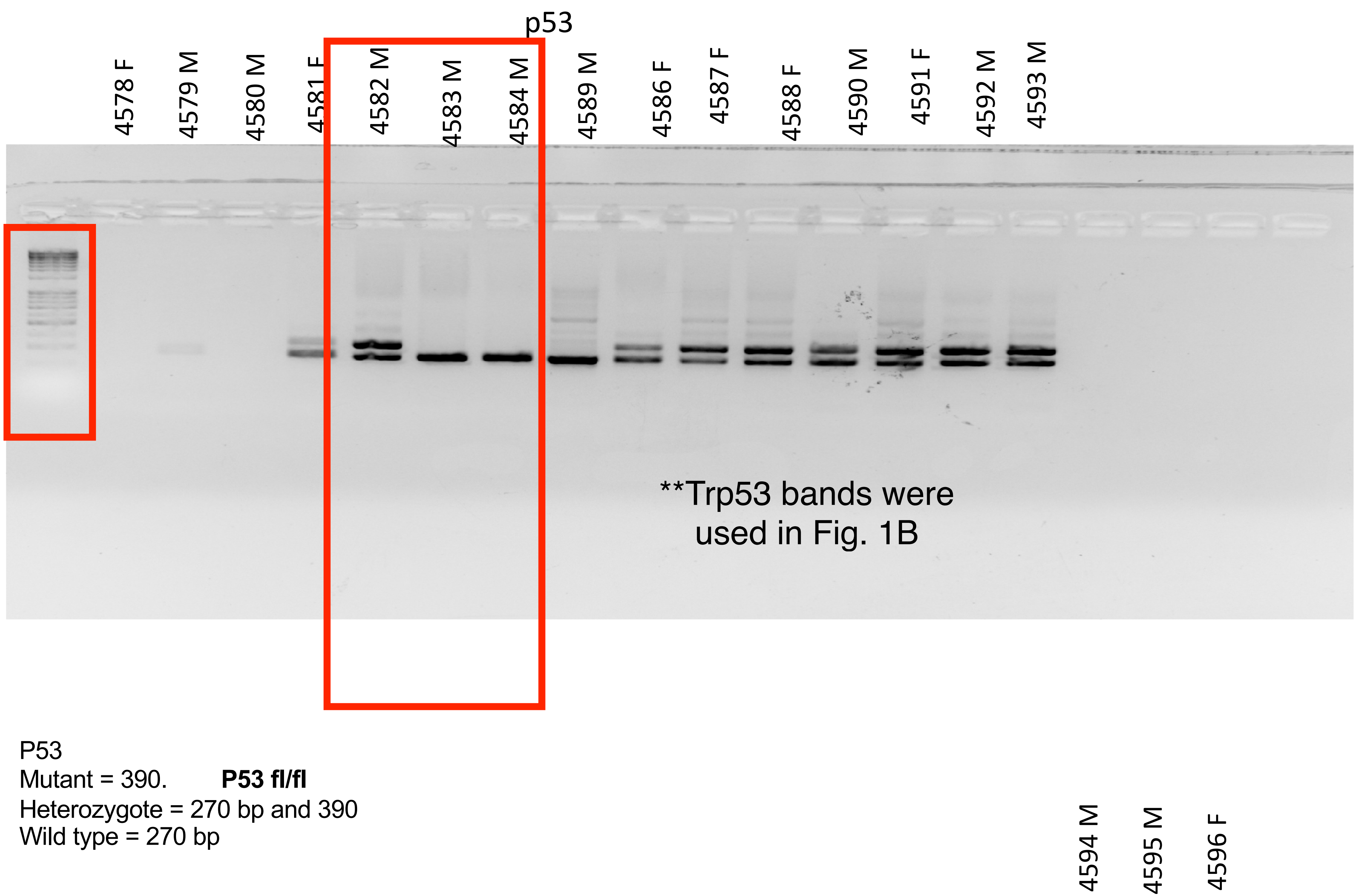

P53
Mutant = 390. **P53 fl/fl**
Heterozygote = 270 bp and 390
Wild type = 270 bp

Litter 4

KP x 2 KPY

**Yap1 bands were used in Fig. 1B

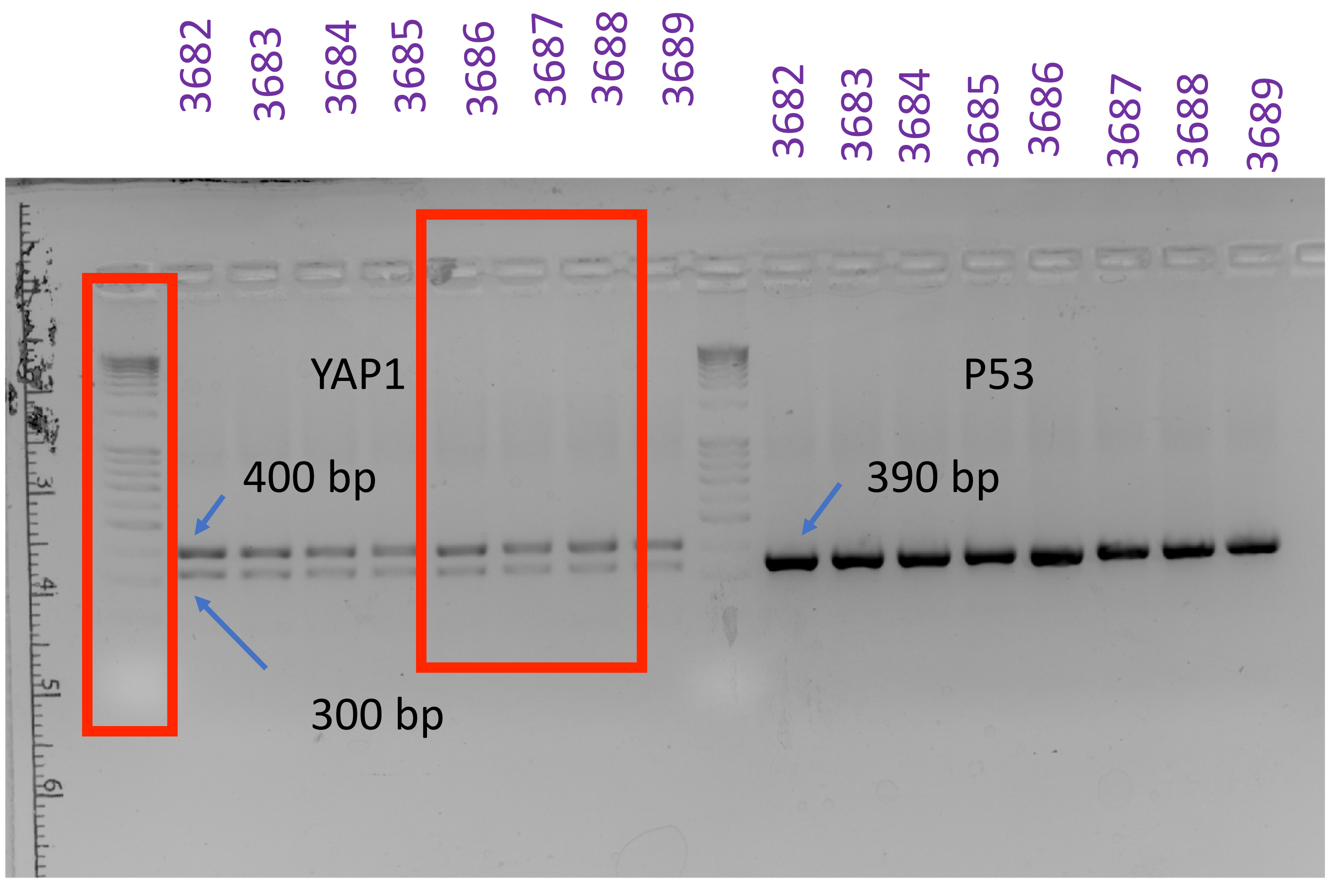

All are Yap1 fl/+ and p53 fl/fl

YAP1 mutant 400 bp
Het 300 +400
WT 300 bp

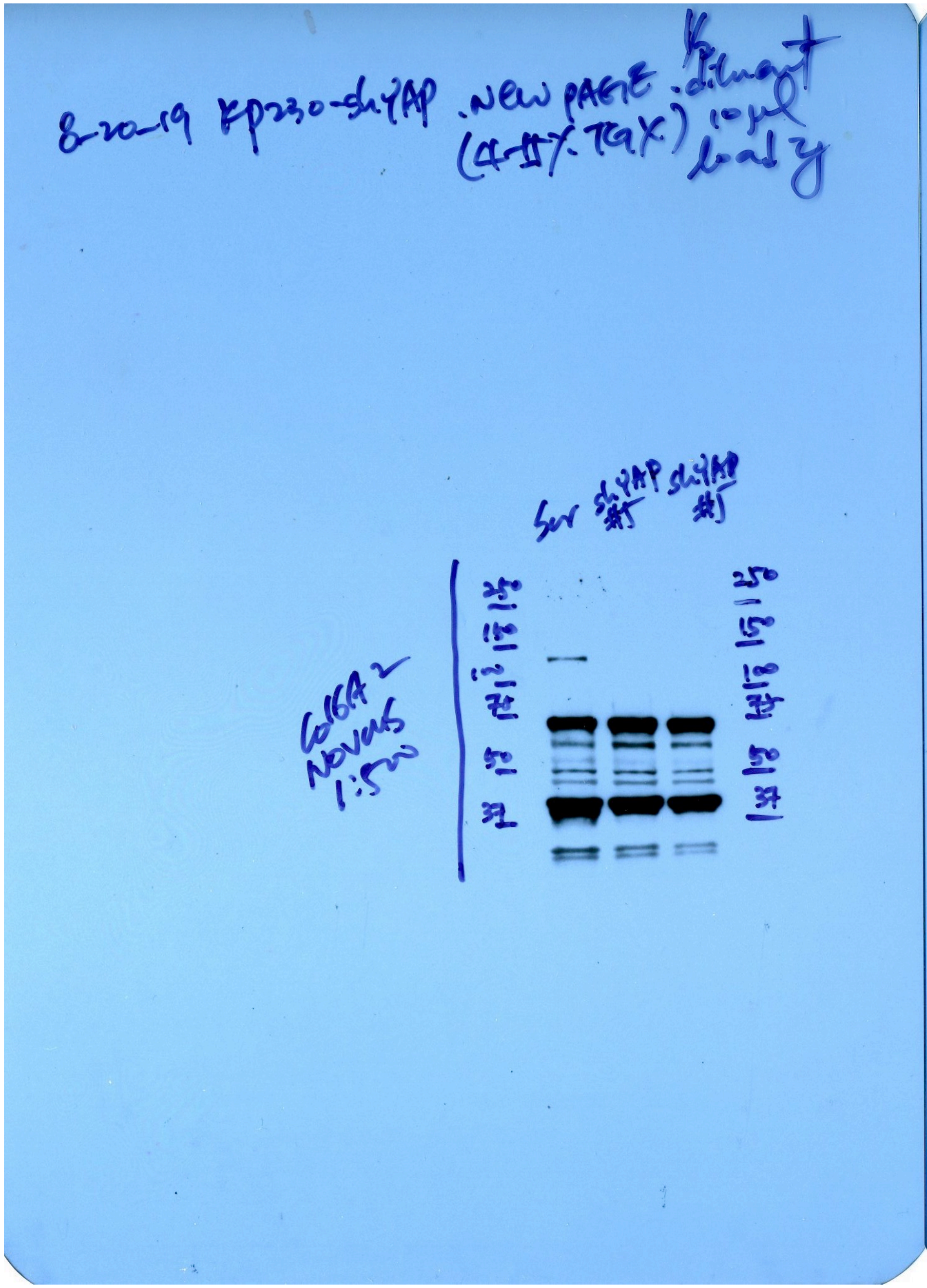

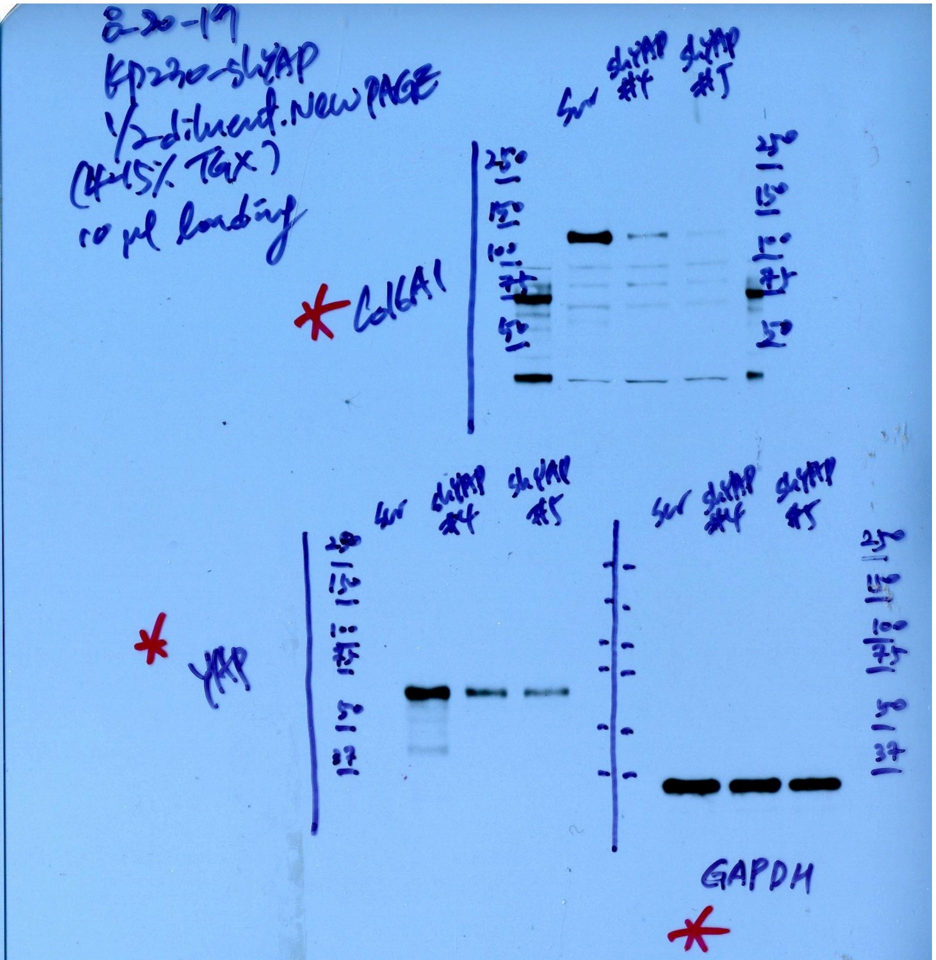

Use the blots w/ red asterisks for Fig. 2E, all three lanes:

Col6a1 = ~150 kDa (note appearance of ladder lines on far left overlapping the handwritten text)

Yap1

Gapdh

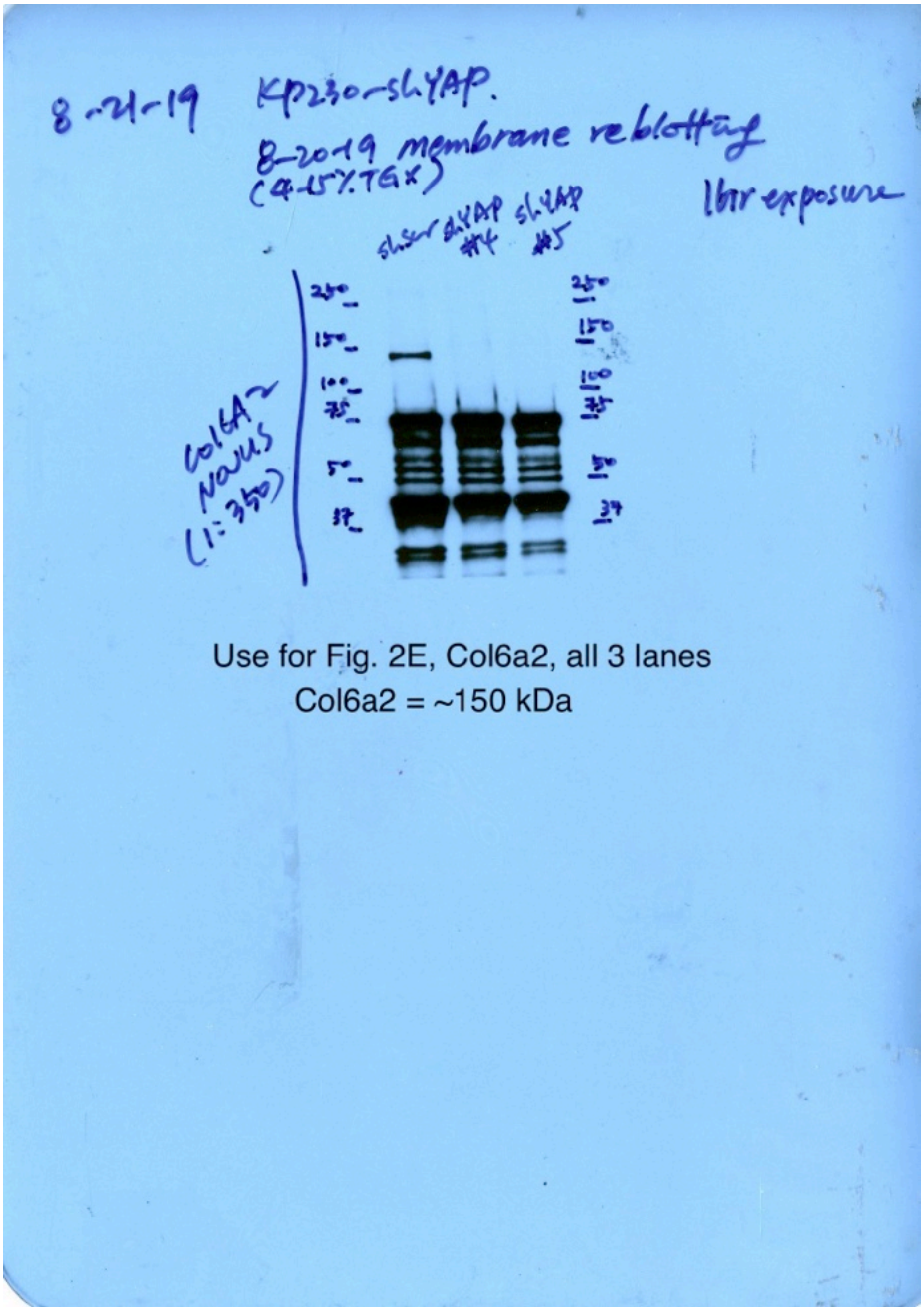

Use for Fig. 2E, Col6a2, all 3 lanes

Col6a2 = ~150 kDa

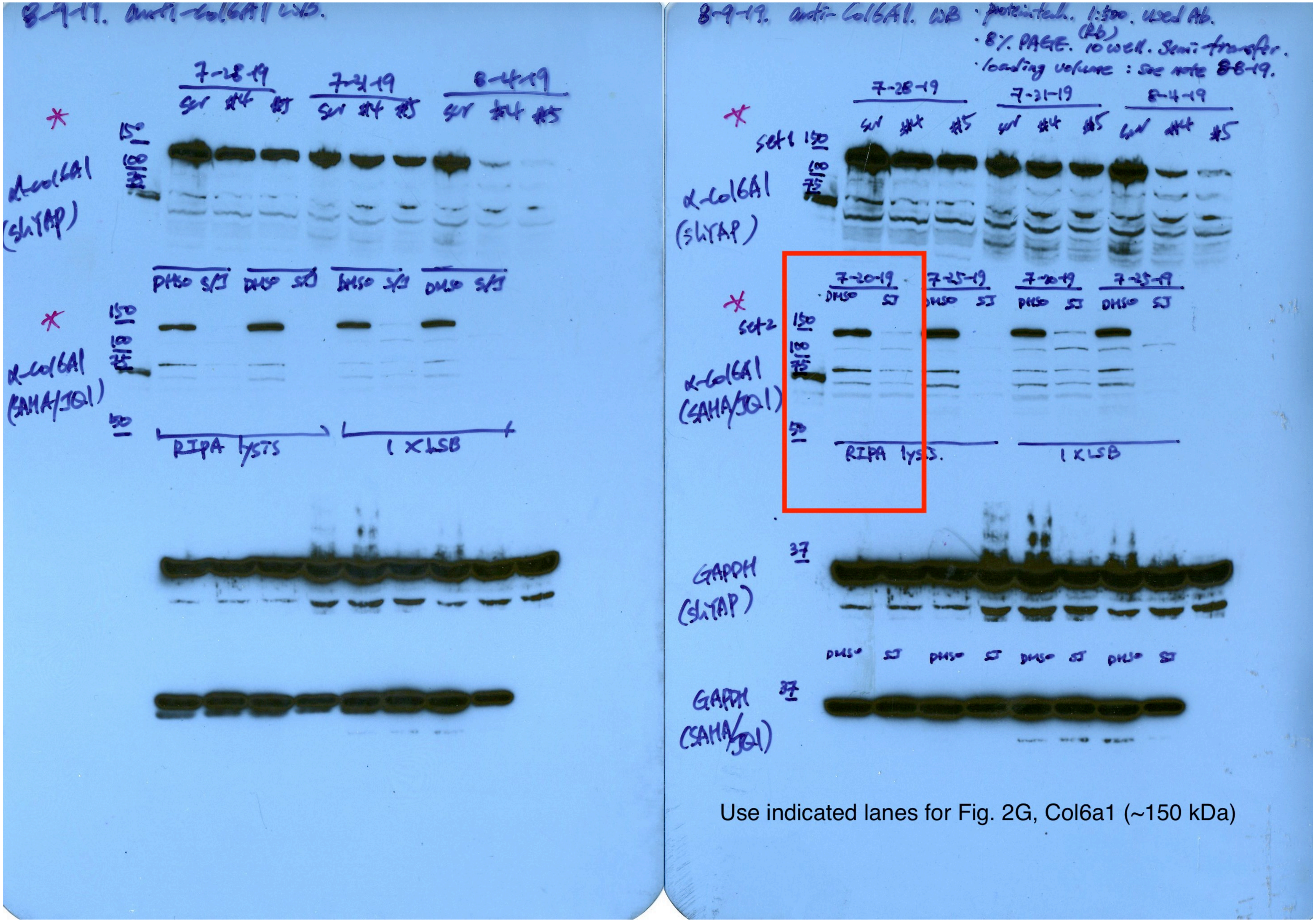

Use indicated lanes for Fig. 2G, Col6a1 (~150 kDa)

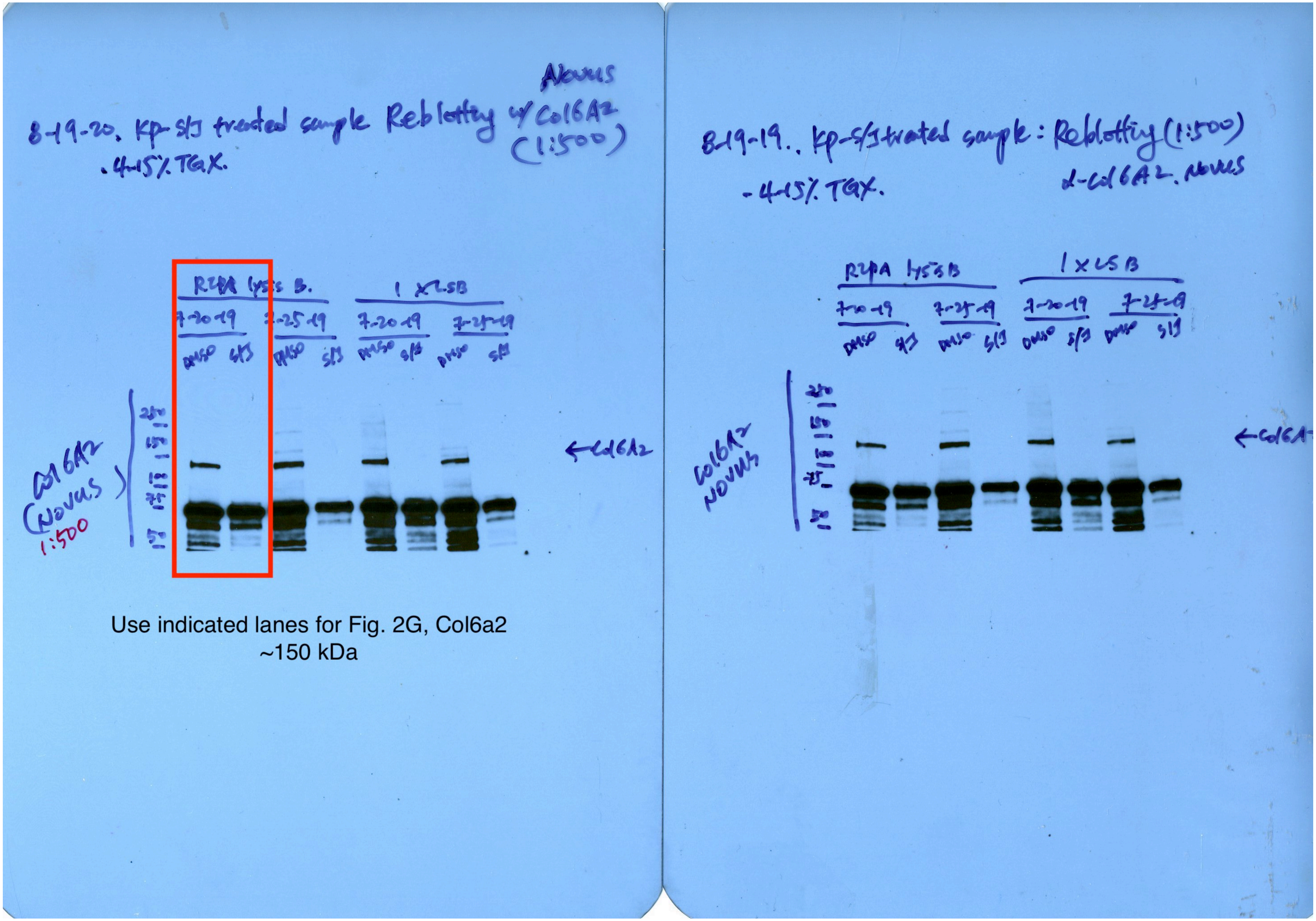

Use indicated lanes for Fig. 2G, Col6a2
~150 kDa

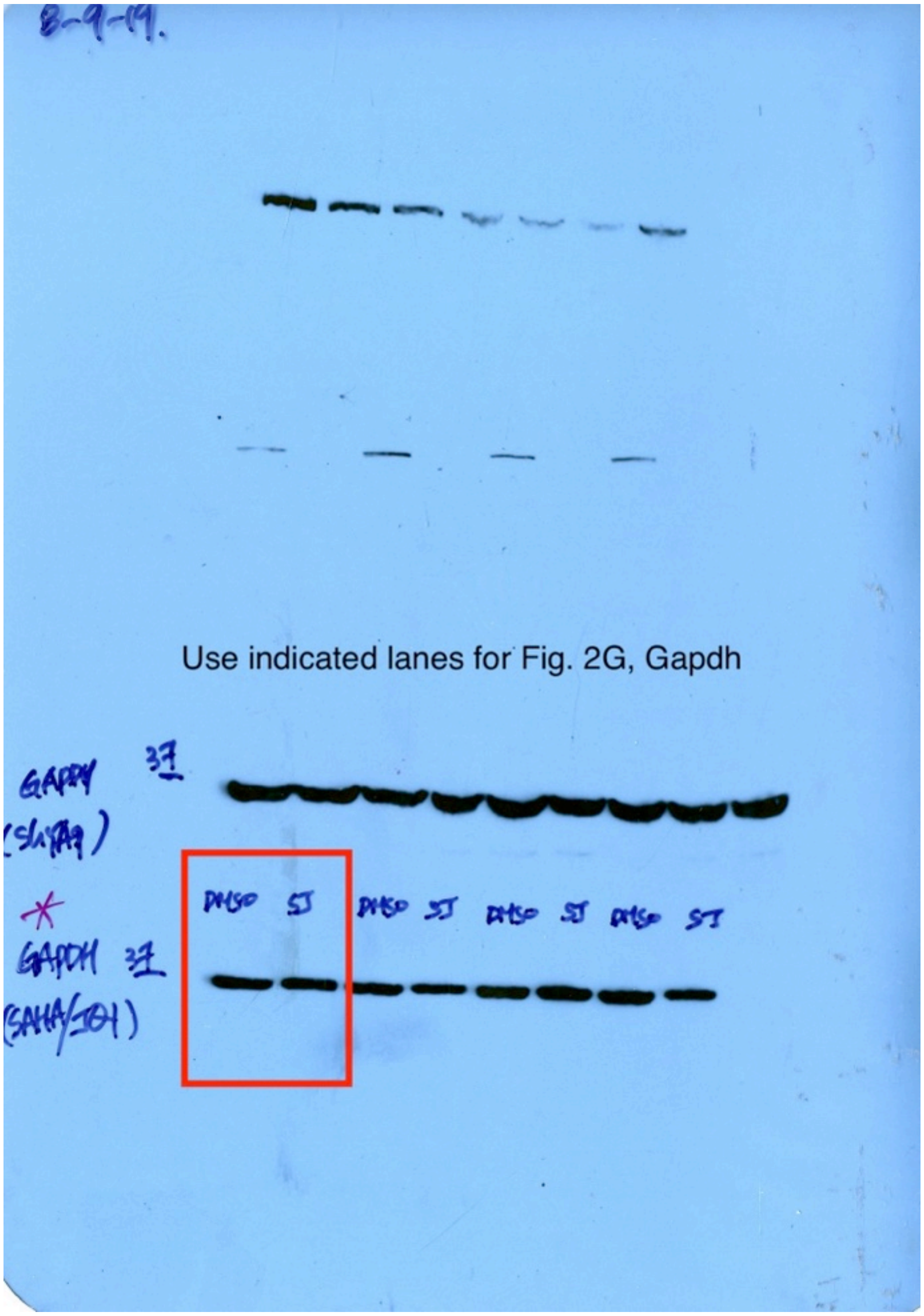

Use indicated lanes for Fig. 2G, Gapdh

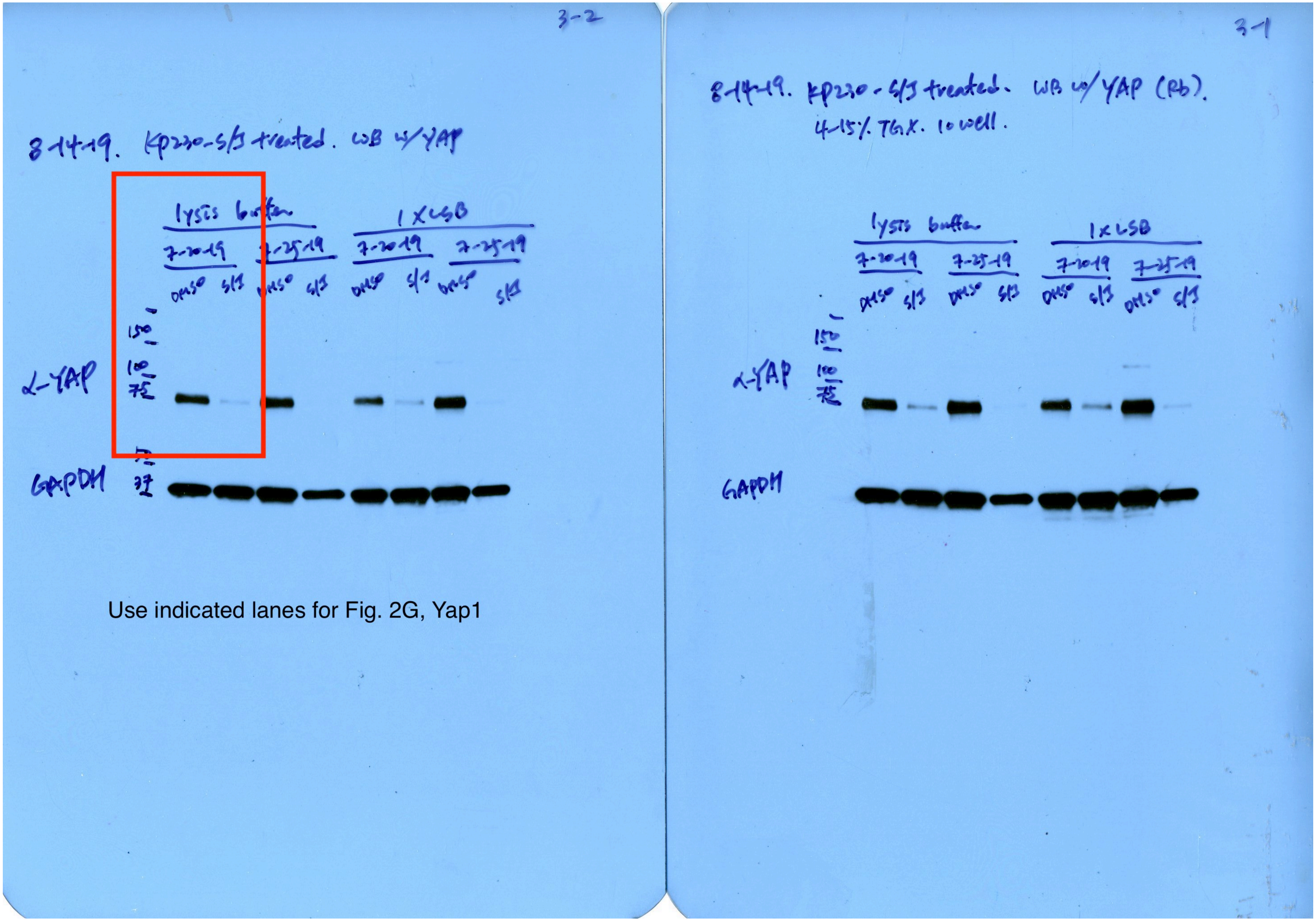

Use indicated lanes for Fig. 2G, Yap1

Fig. 6D, Gapdh

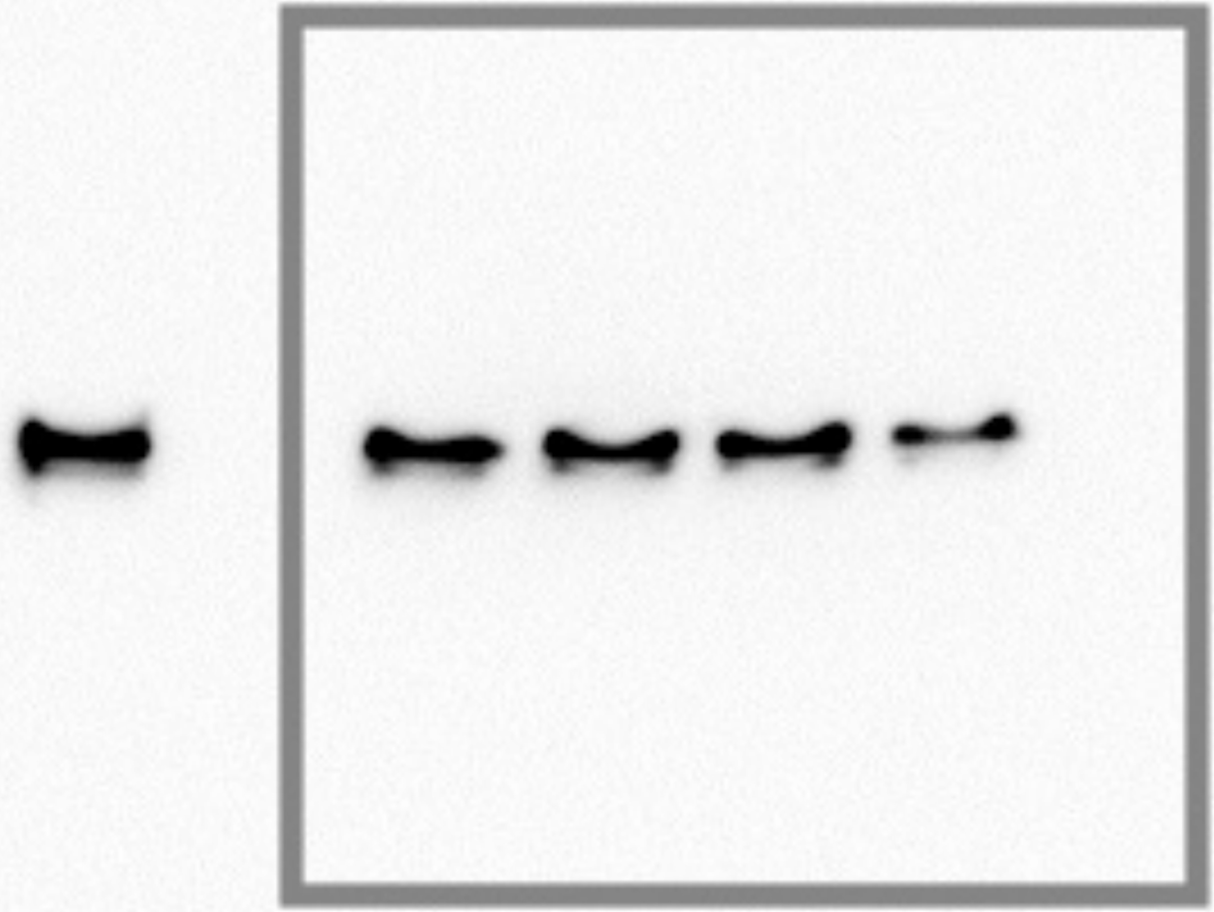

This blot is from the same membrane as the LC3B blot below. The membrane was cut and the two sections were imaged separately because very different exposure times were needed.

Fig. 6D Lc3b

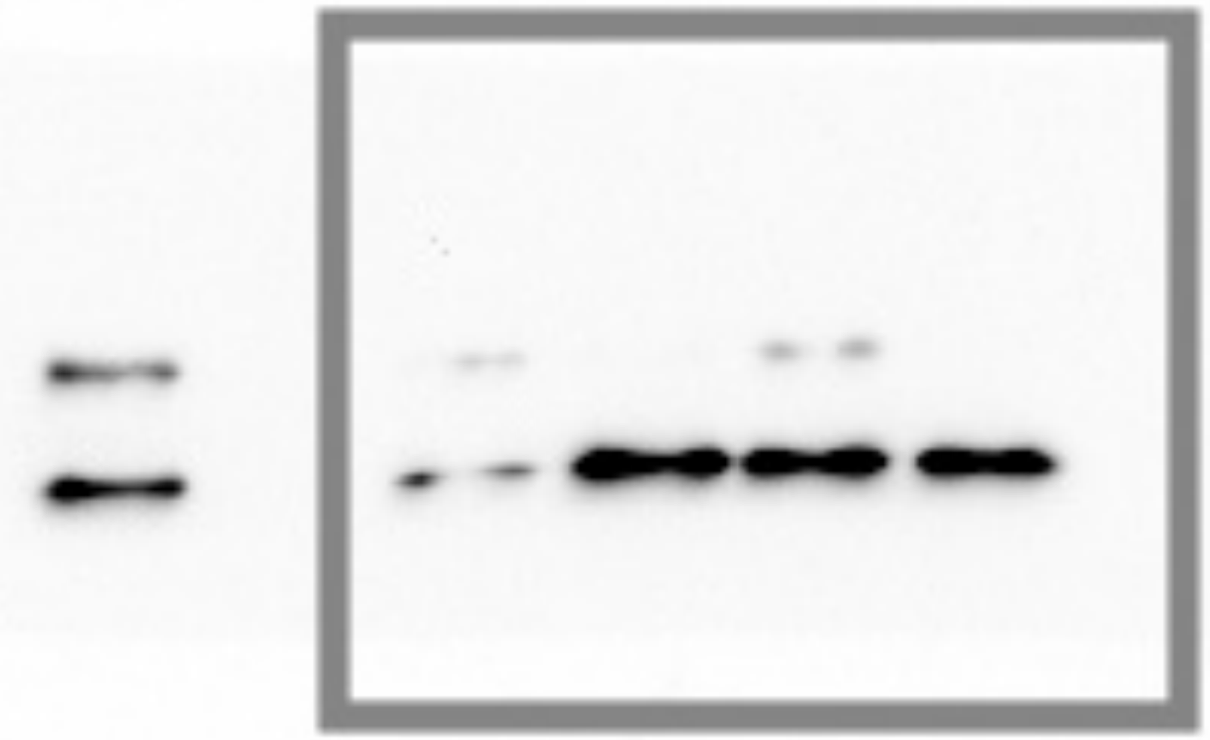

This blot is from the same membrane as the GAPDH blot above. The membrane was cut and the two sections were imaged separately because very different exposure times were needed.

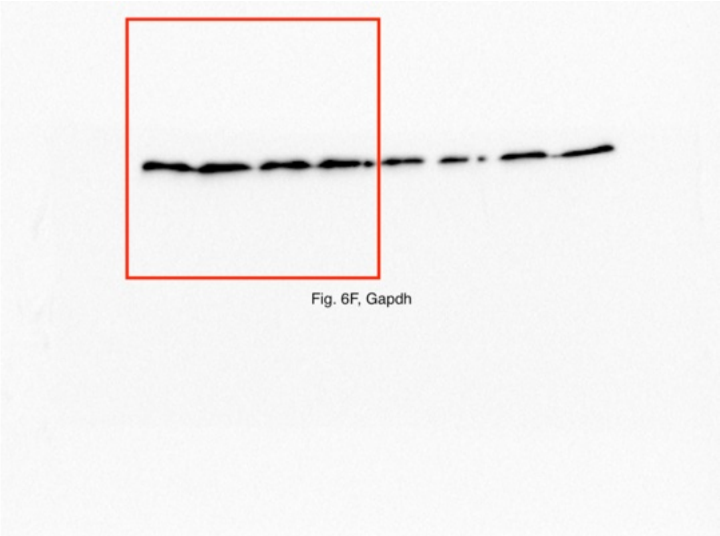

Fig. 6F, Gapdh

Fig. 6F, LC3B
All 4 lanes

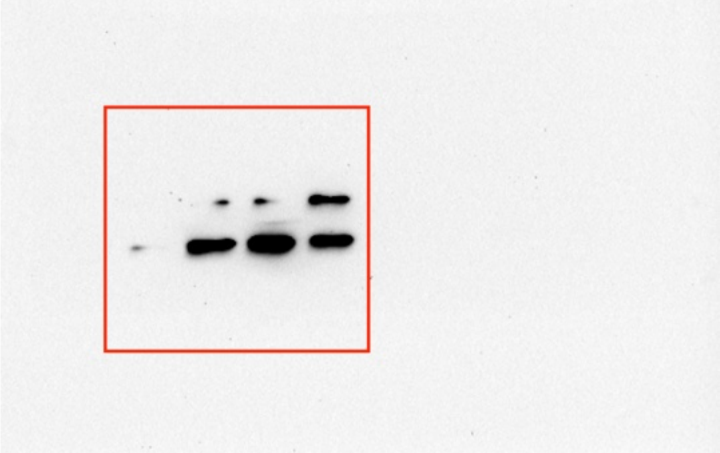

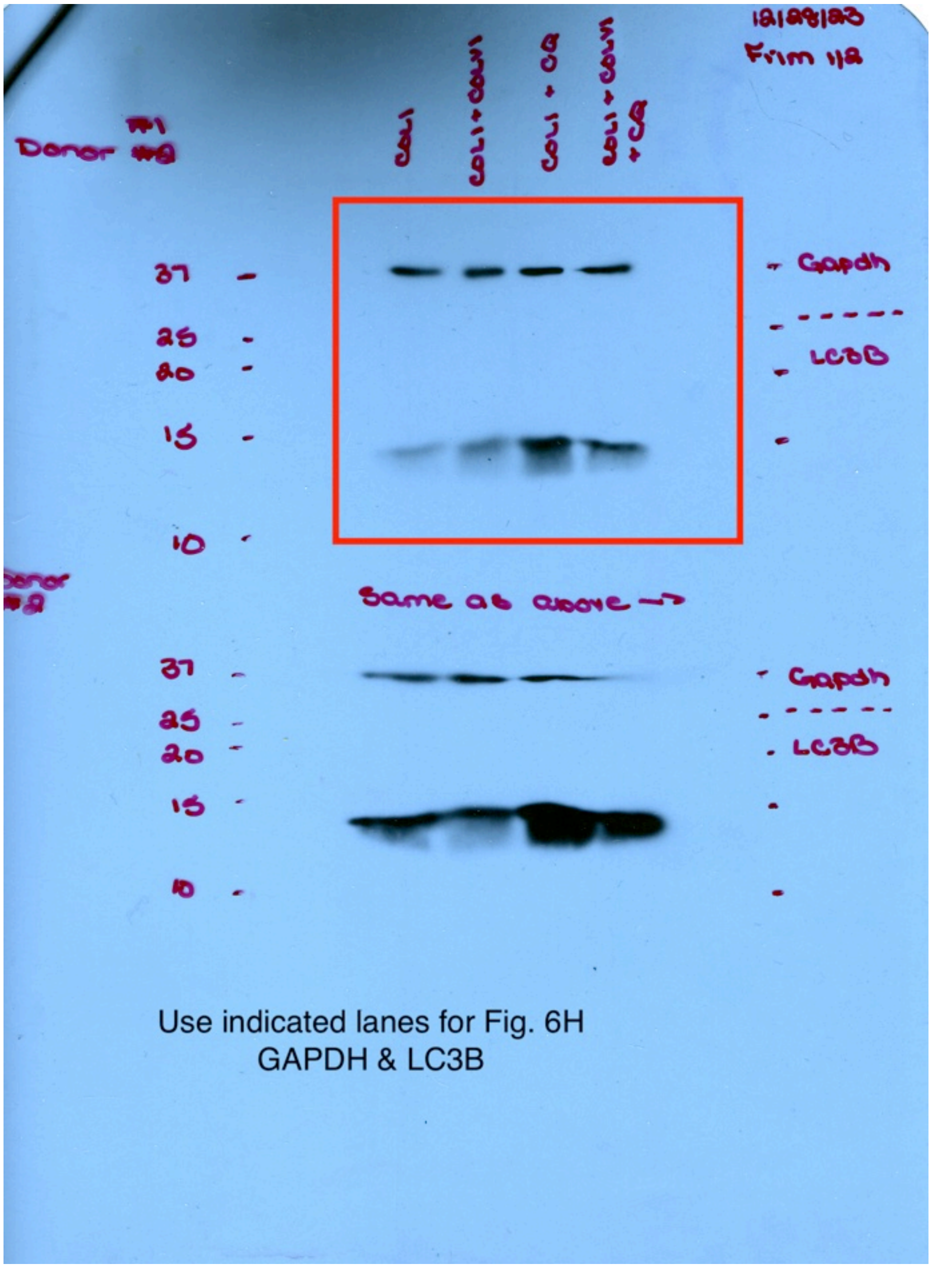

Use indicated lanes for Fig. 6H
GAPDH & LC3B

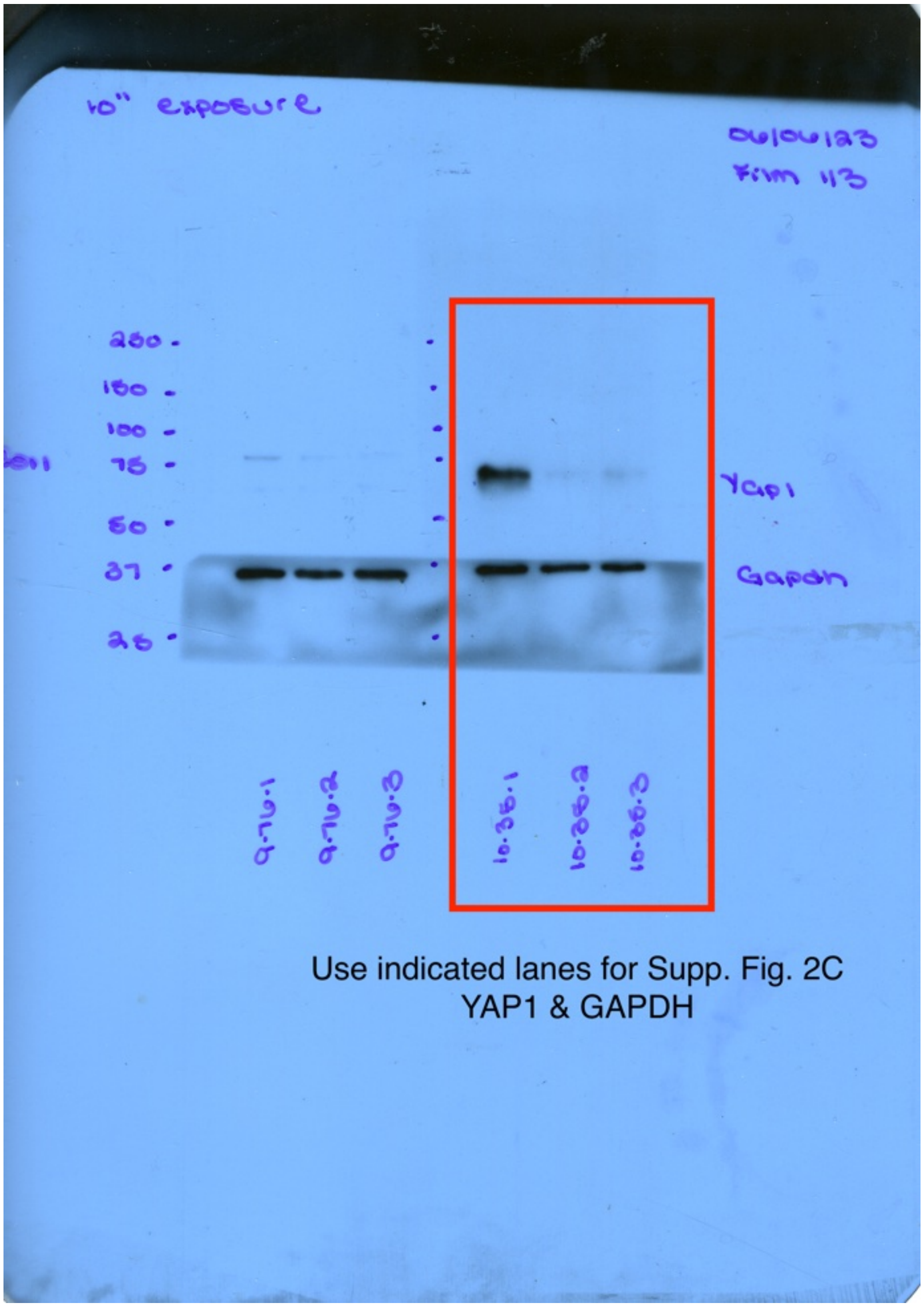

Use indicated lanes for Supp. Fig. 2C
YAP1 & GAPDH

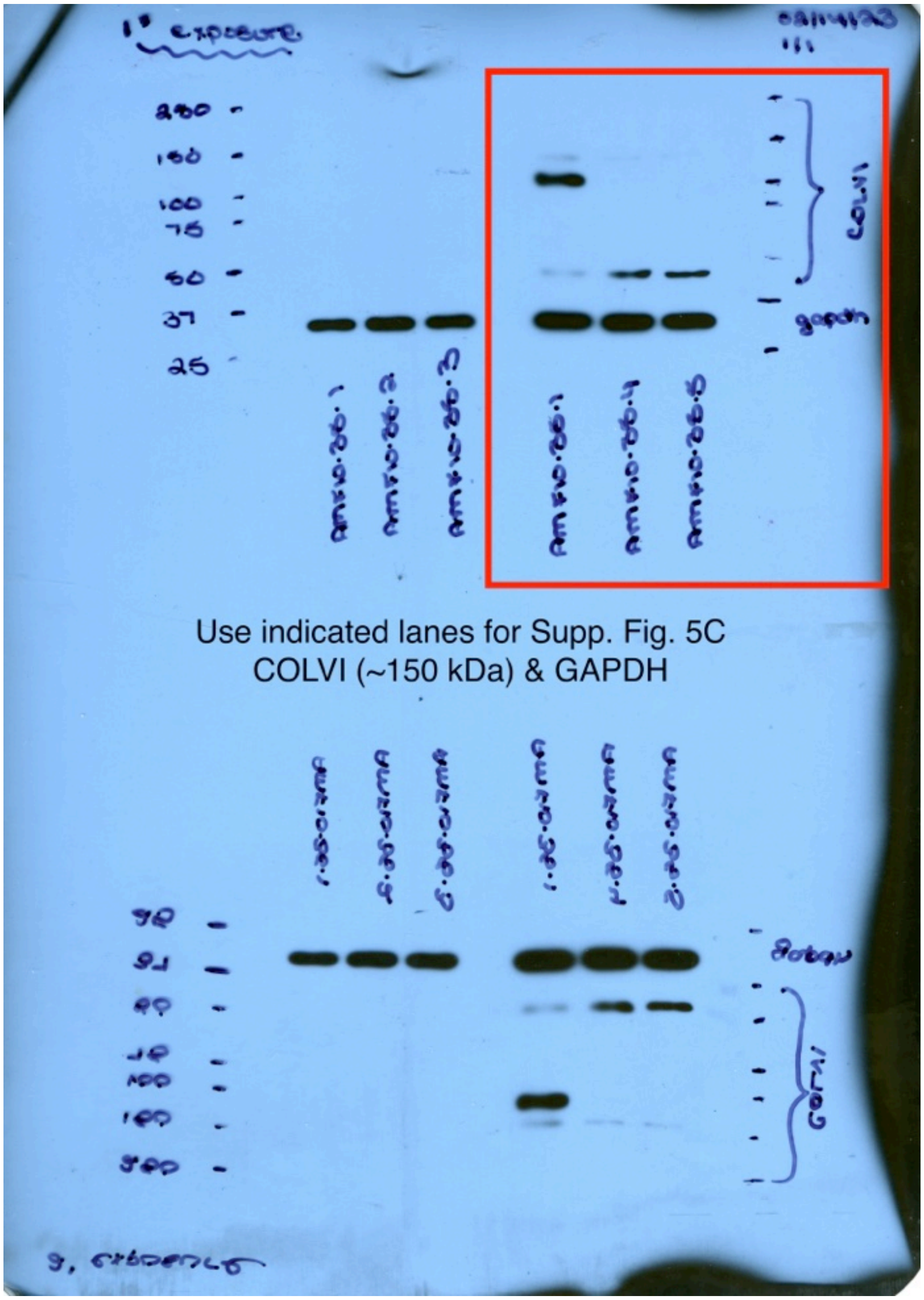

Use indicated lanes for Supp. Fig. 5C
COLVI (~150 kDa) & GAPDH

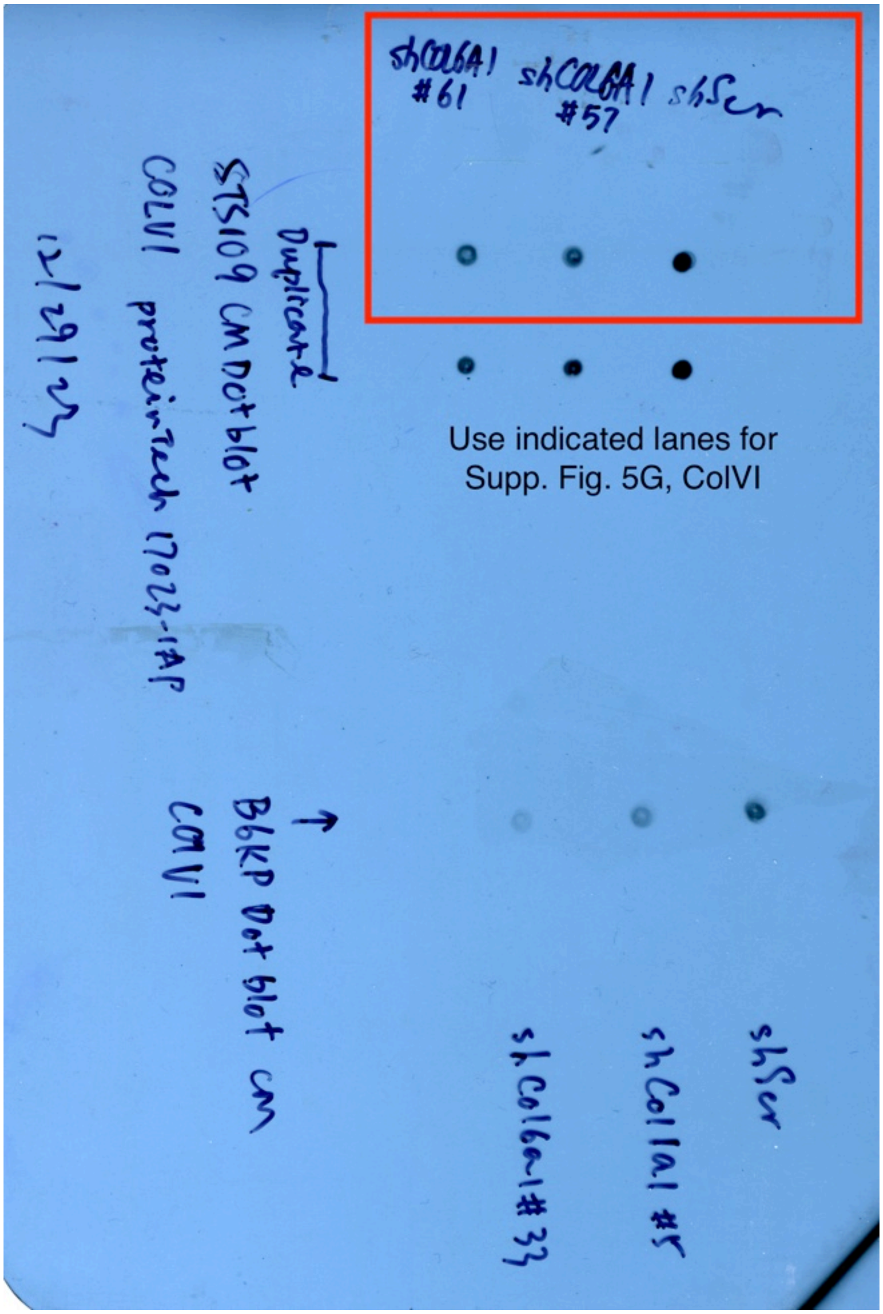
shCOL6A1 #61
shCOL6A1 #57
shScr
Use indicated lanes for Supp. Fig. 5G, ColVI
Duplicate
STS109 CM Dot blot
ColVI proteinTech 17023-1AP
12/29/23
B6KP Dot blot CM
ColVI
shScr
shCol1a1 #5
shCol6a1 #33

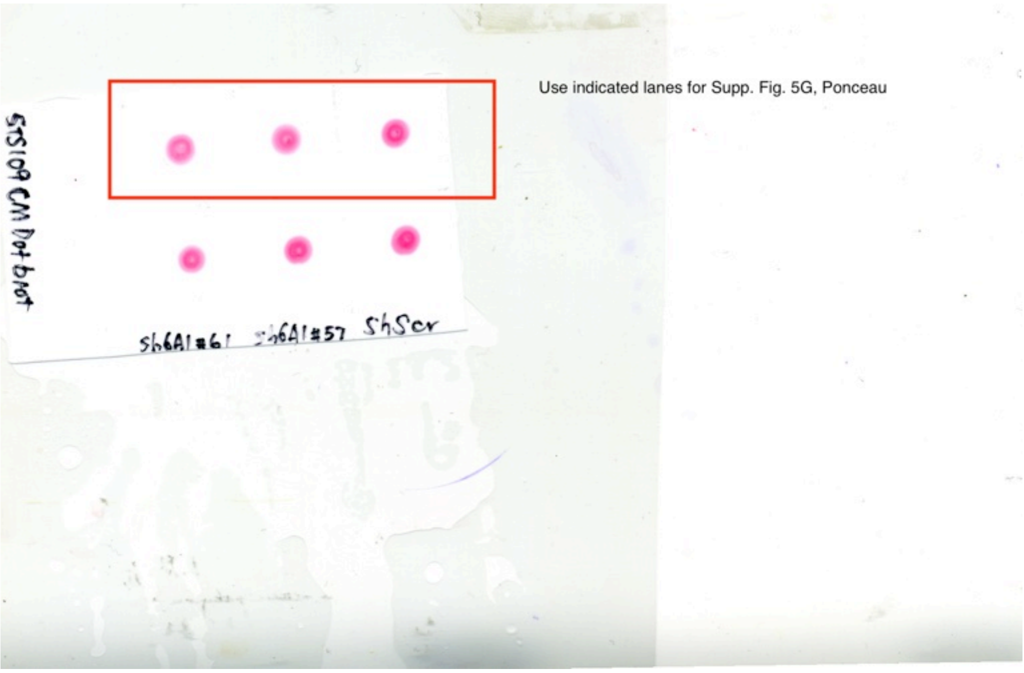

Use indicated lanes for Supp. Fig. 5G, Ponceau

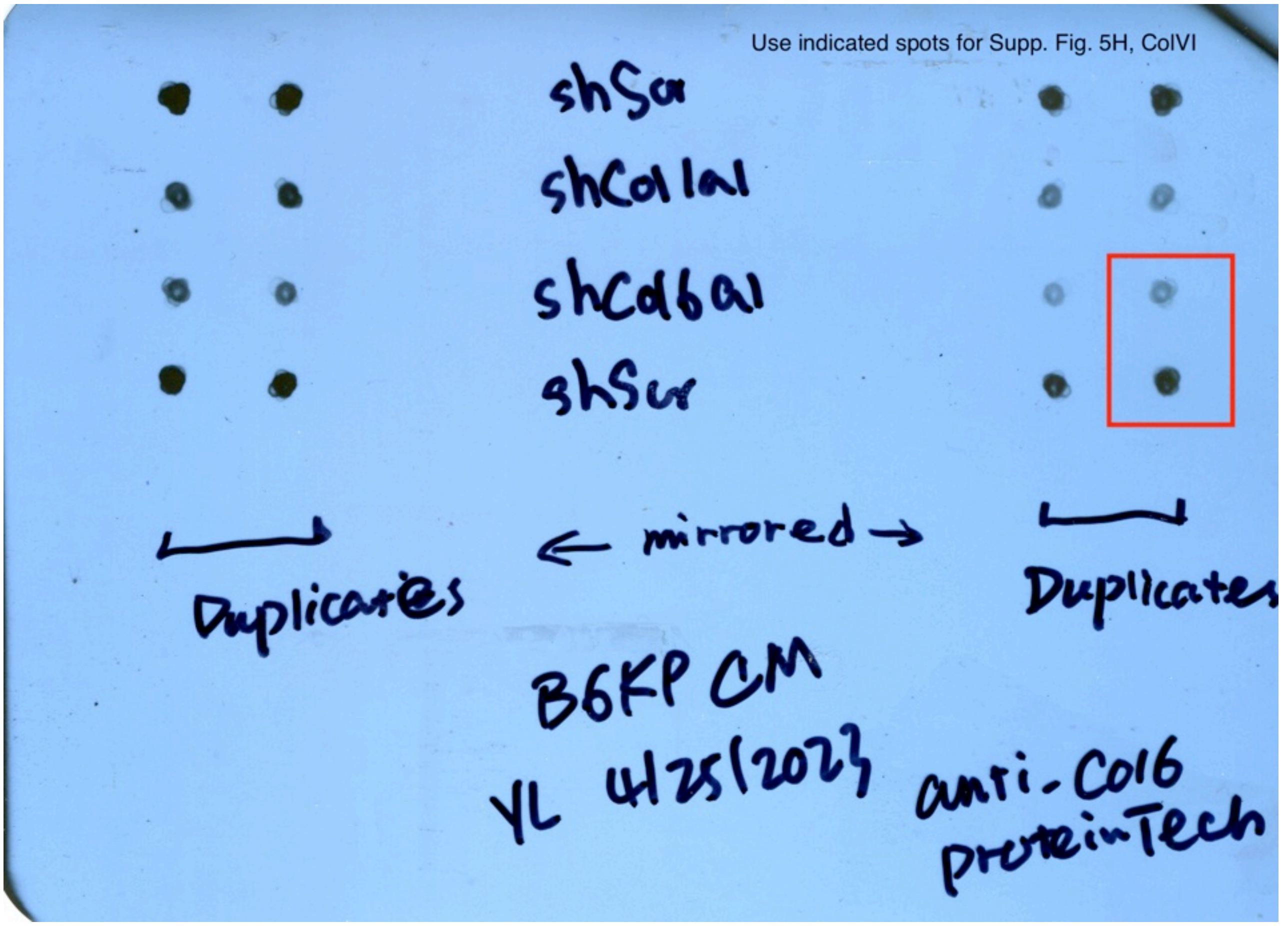
Use indicated spots for Supp. Fig. 5H, ColVI
shScr
shCol1a1
shCol6a1
shScr
Duplicates
mirrored
Duplicates
B6KP CM
YL 4/25/2023
anti-Col6
proteinTech

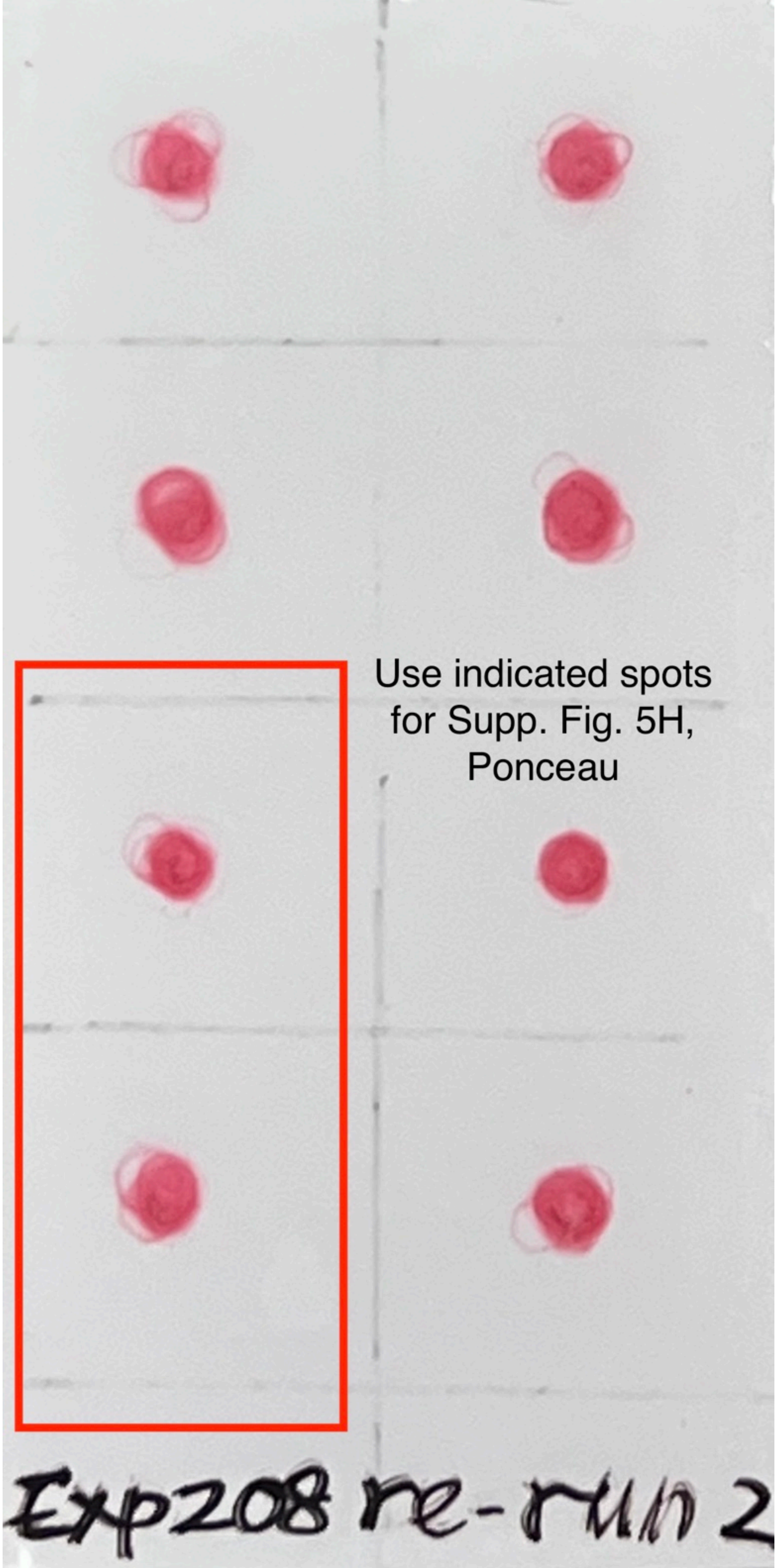

Use indicated spots for Supp. Fig. 5H, Ponceau

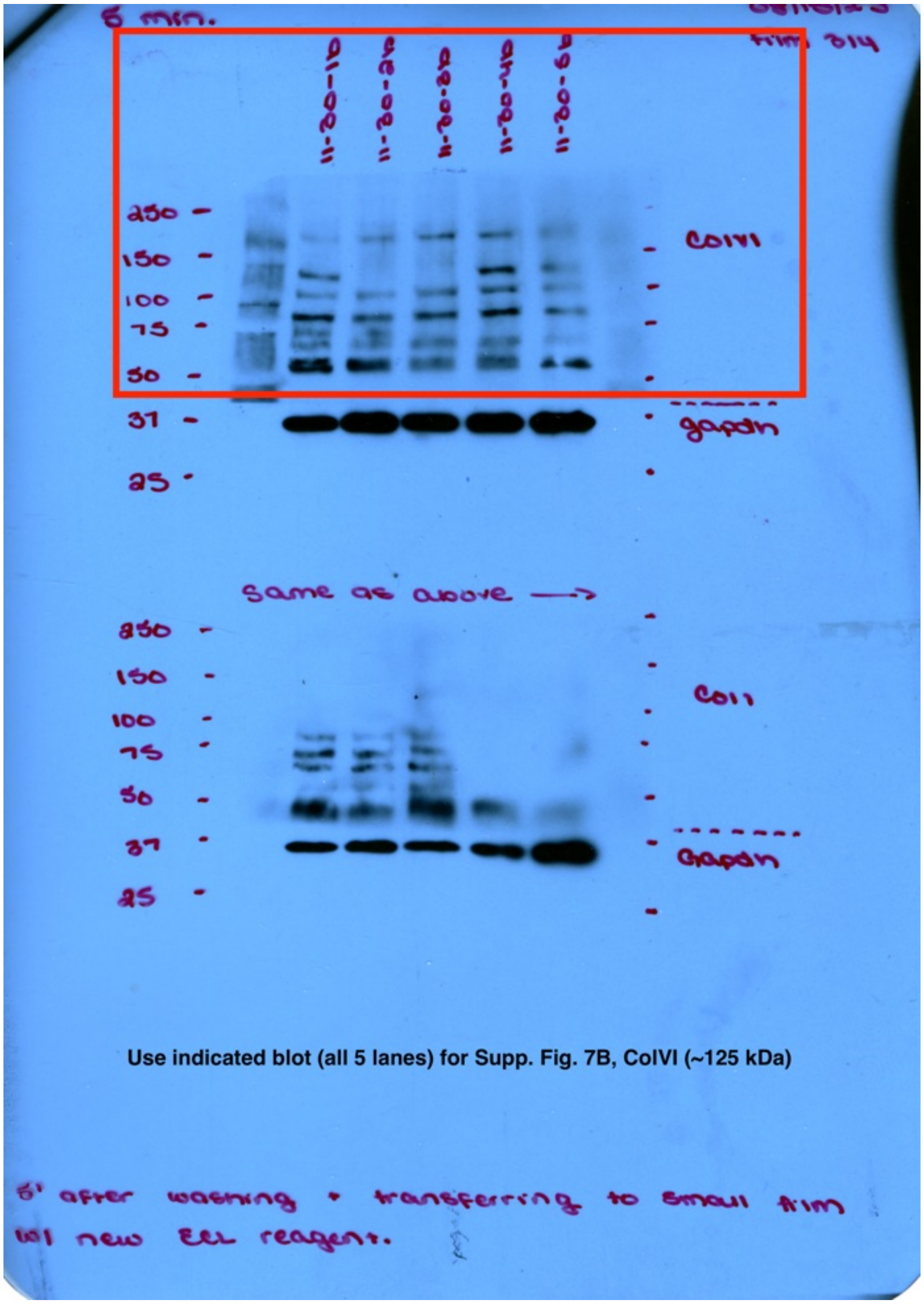

Use indicated blot (all 5 lanes) for Supp. Fig. 7B, ColVI (~125 kDa)

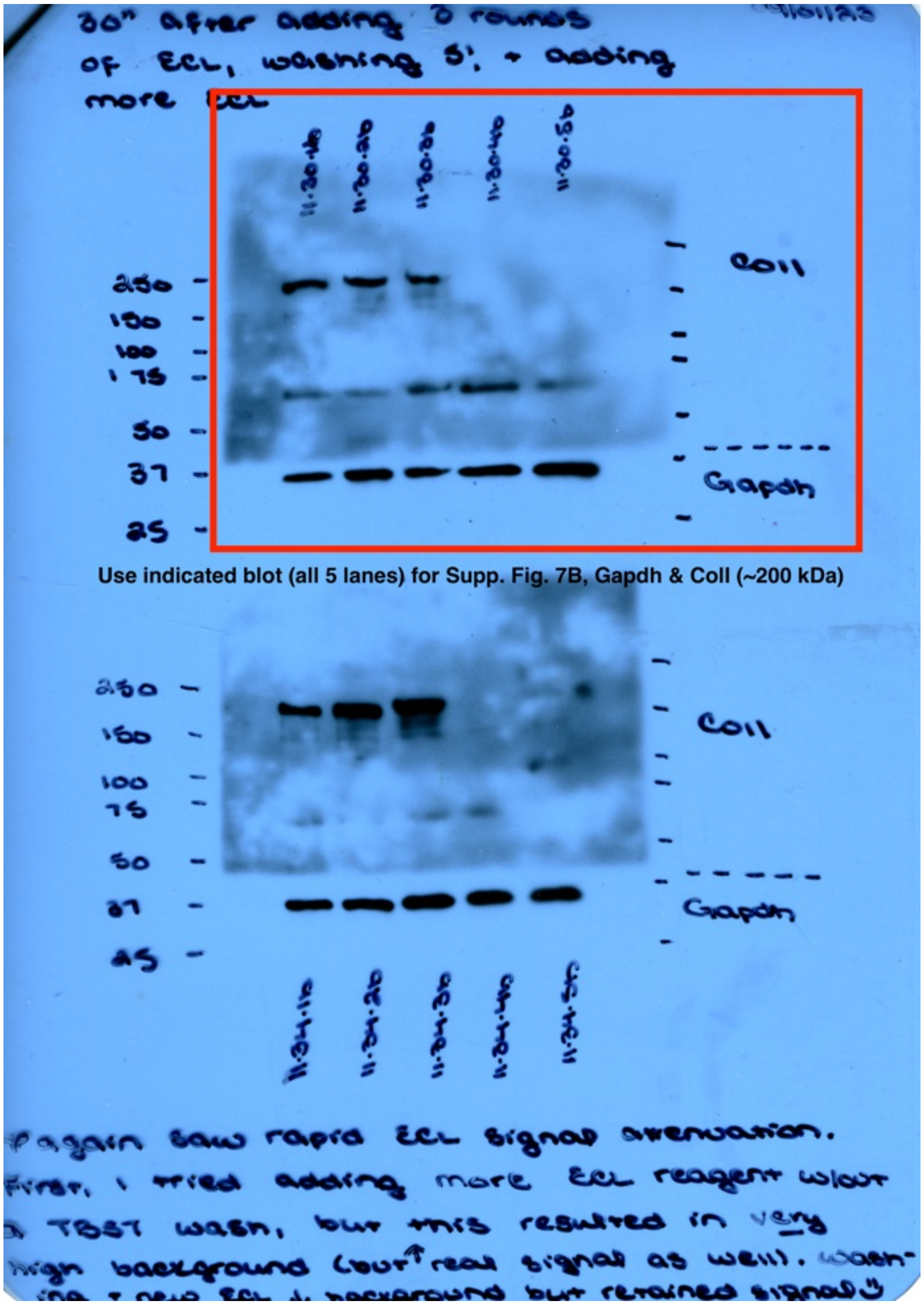

Use indicated blot (all 5 lanes) for Supp. Fig. 7B, Gapdh & Coll (~200 kDa)

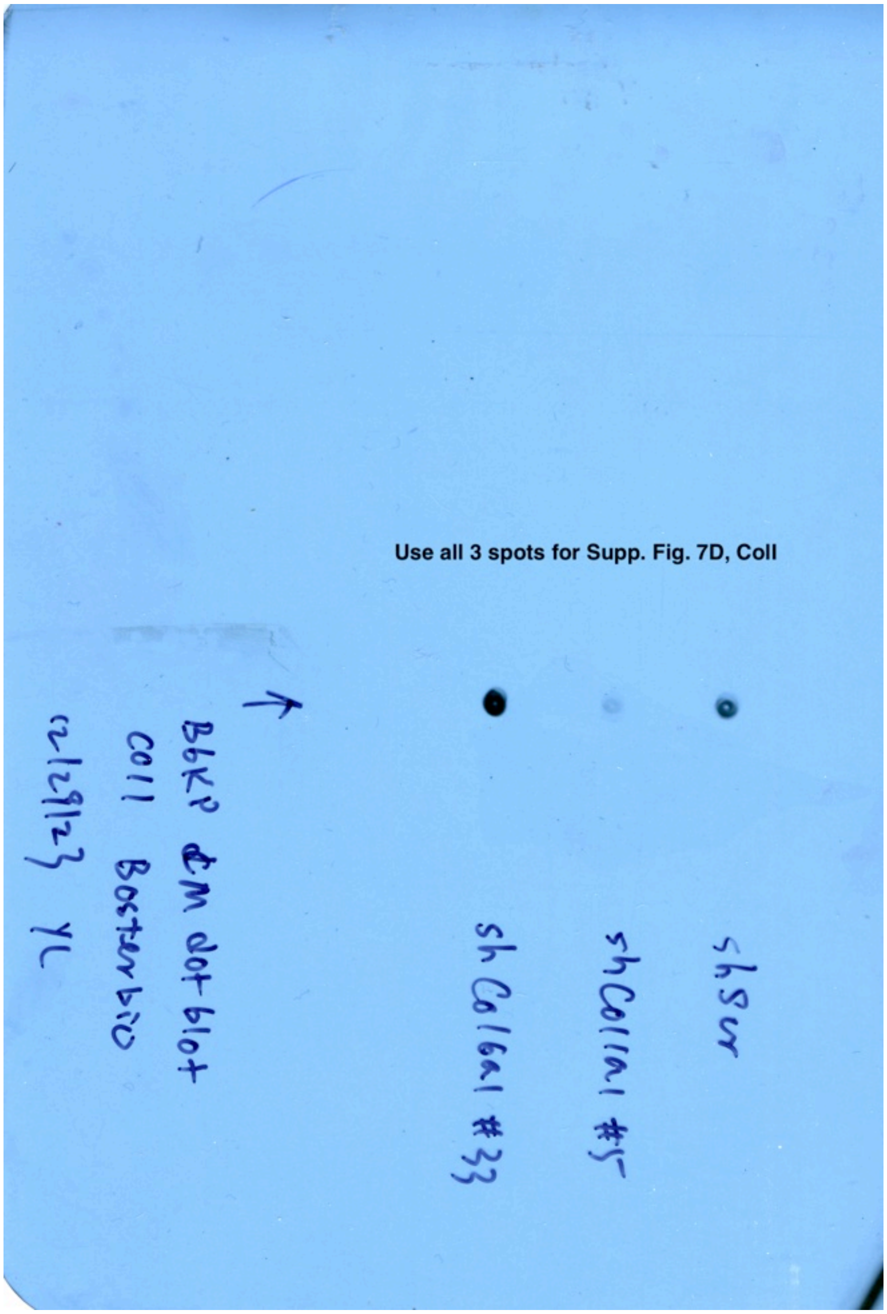

Use all 3 spots for Supp. Fig. 7D, Coll

Use indicated spots for Supp. Fig. 7D, Ponceau

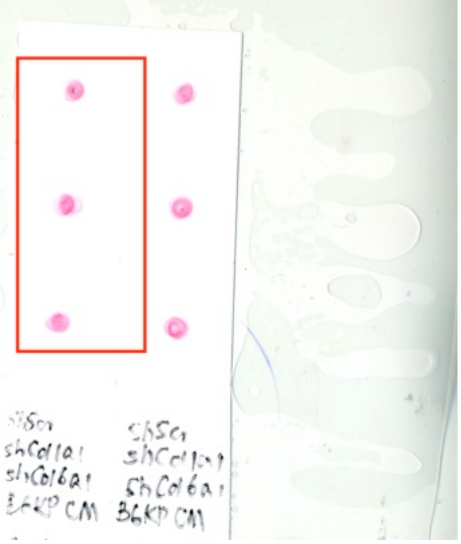

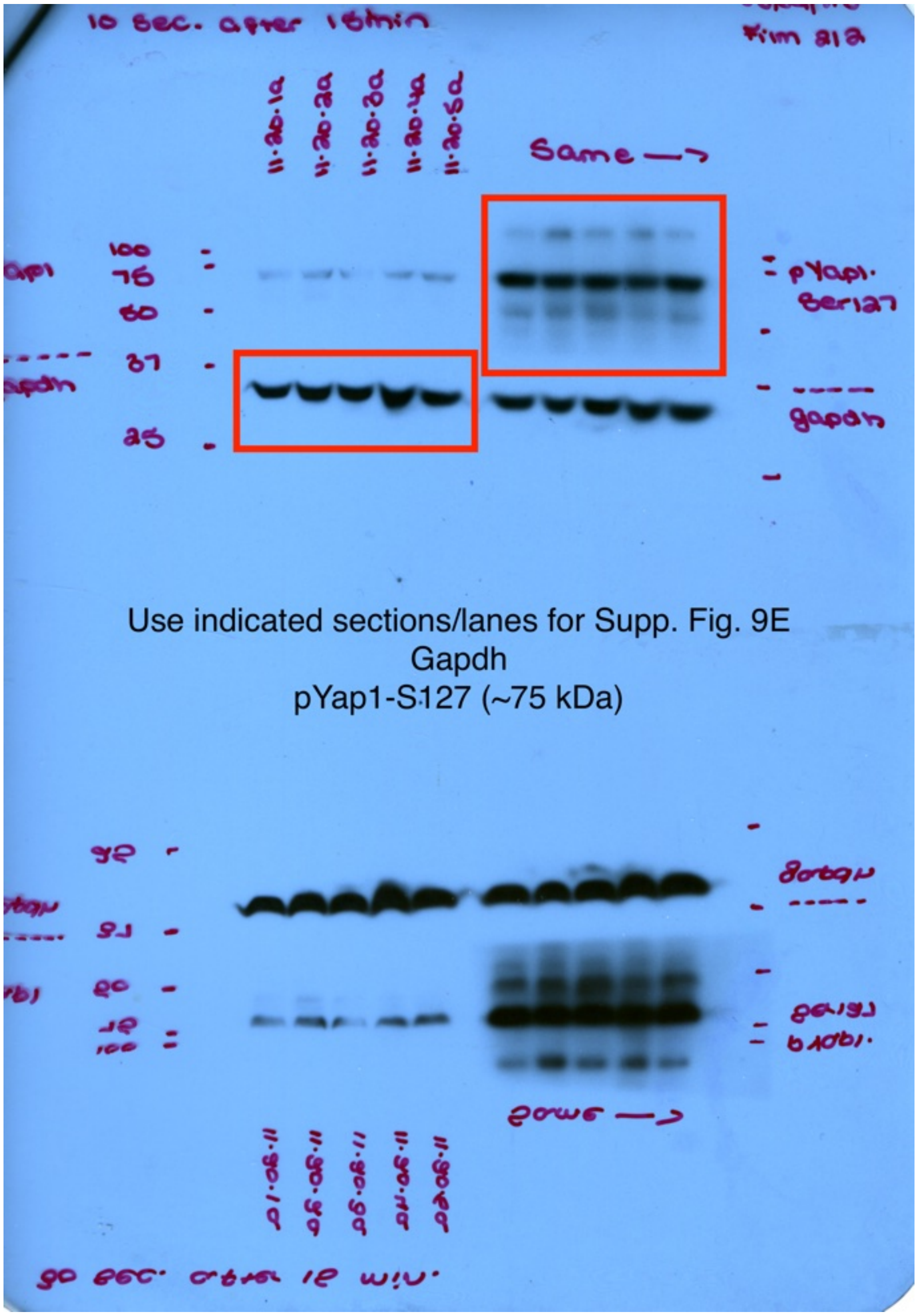

Use indicated sections/lanes for Supp. Fig. 9E
Gapdh
pYap1-S127 (~75 kDa)

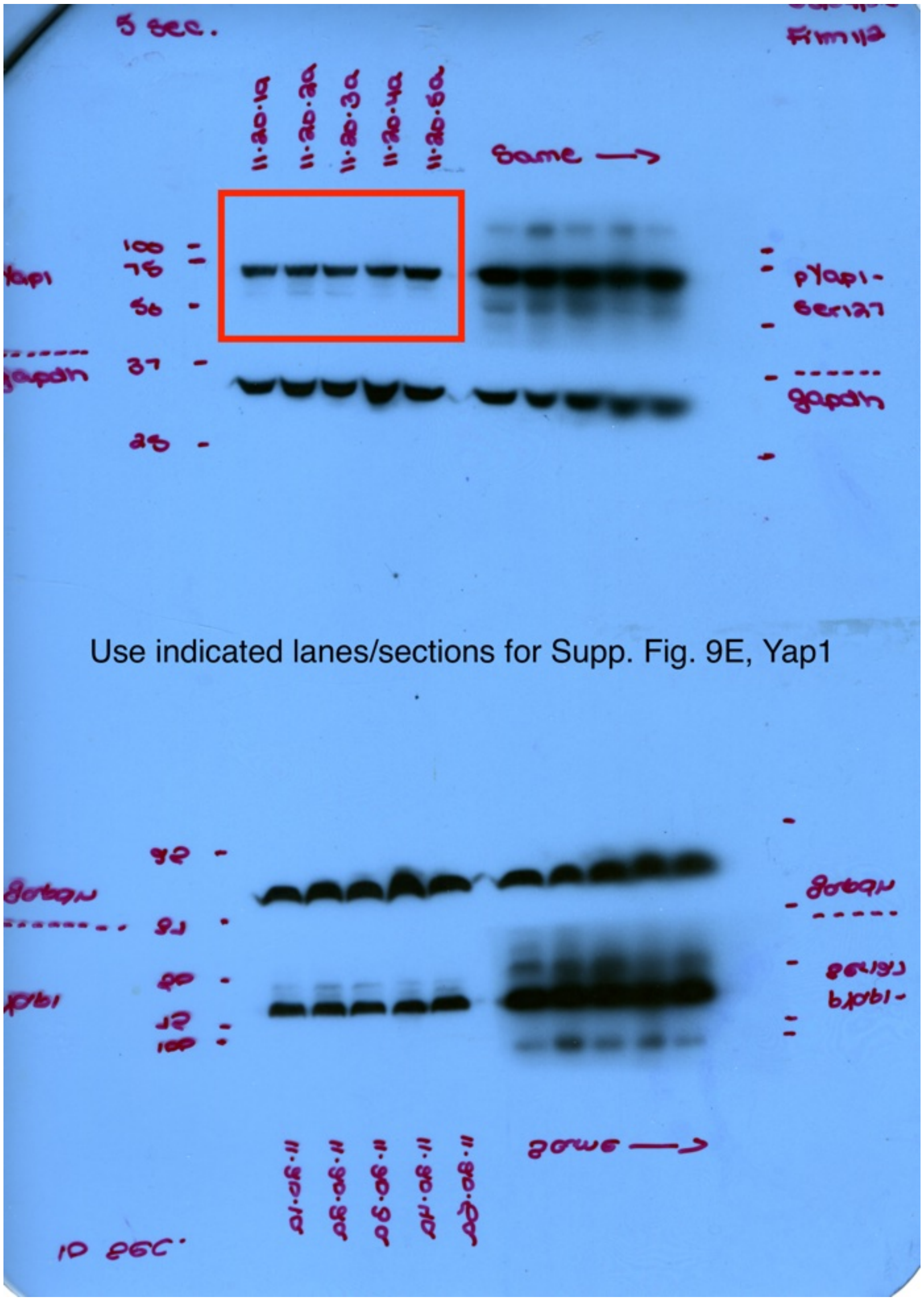

Use indicated lanes/sections for Supp. Fig. 9E, Yap1

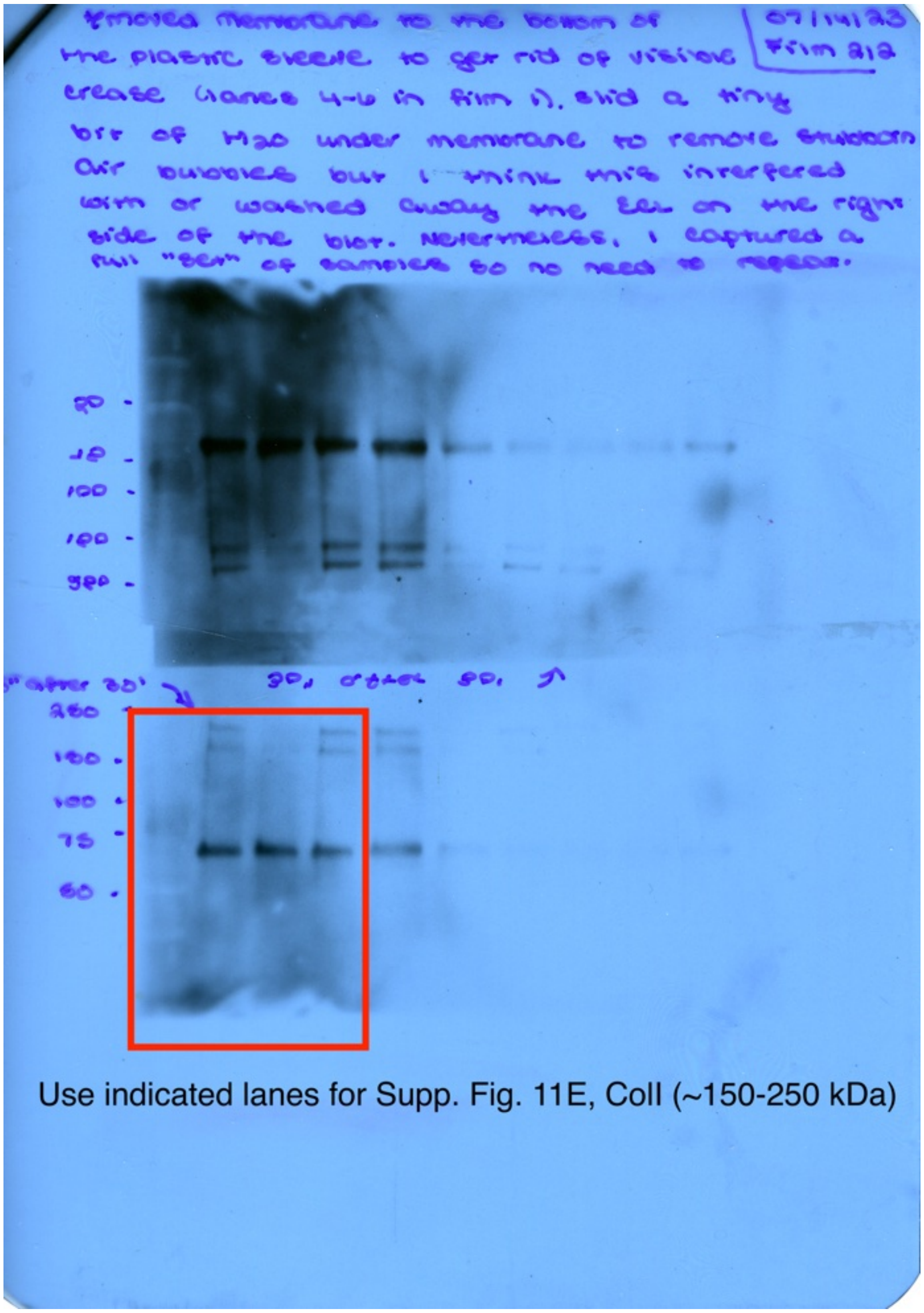

Use indicated lanes for Supp. Fig. 11E, ColI (~150-250 kDa)
